# Postglacial phylogeography, admixture, and evolution of red spruce (*Picea rubens* Sarg.) in Eastern North America

**DOI:** 10.3389/fpls.2023.1272362

**Published:** 2023-10-12

**Authors:** Stanislav Bashalkhanov, Jeremy S. Johnson, Om P. Rajora

**Affiliations:** ^1^ Faculty of Forestry and Environmental Management, University of New Brunswick, Fredericton, NB, Canada; ^2^ Department of Forestry, Michigan State University, East Lansing, MI, United States

**Keywords:** postglacial migration, glacial refugia, genetic diversity and population structure, interspecific hybridization, molecular evolution, approximate Bayesian computation, biogeography

## Abstract

Climate change is a major evolutionary force that can affect the structure of forest ecosystems worldwide. Red spruce (*Picea rubens* Sarg.) has recently faced a considerable decline in the Southern Appalachians due to rapid environmental change, which includes historical land use, and atmospheric pollution. In the northern part of its range, red spruce is sympatric with closely related black spruce (*Picea mariana* (Mill.) B.S.P.), where introgressive hybridization commonly occurs. We investigated range-wide population genetic diversity and structure and inferred postglacial migration patterns and evolution of red spruce using nuclear microsatellites. Moderate genetic diversity and differentiation were observed in red spruce. Genetic distance, maximum likelihood and Bayesian analyses identified two distinct population clusters: southern glacial populations, and the evolutionarily younger northern populations. Approximate Bayesian computation suggests that patterns of admixture are the result of divergence of red spruce and black spruce from a common ancestor and then introgressive hybridization during post-glacial migration. Genetic diversity, effective population size (*N_e_*) and genetic differentiation were higher in the northern than in the southern populations. Our results along with previously available fossil data suggest that *Picea rubens* and *Picea mariana* occupied separate southern refugia during the last glaciation. After initial expansion in the early Holocene, these two species faced a period of recession and formed a secondary coastal refugium, where introgressive hybridization occurred, and then both species migrated northward. As a result, various levels of black spruce alleles are present in the sympatric red spruce populations. Allopatric populations of *P. rubens* and *P. mariana* have many species-specific alleles and much fewer alleles from common ancestry. The pure southern red spruce populations may become critically endangered under projected climate change conditions as their ecological niche may disappear.

## Introduction

The current structure of forest ecosystems in the Northern Hemisphere formed several thousand years ago because of major transcontinental migrations caused by climate fluctuations after the Last Glacial Maximum (LGM). This is a relatively short time on the evolutionary scale, especially for long-lived tree species, whose frontier populations may have occupied their current habitats for only a small number of generations. Repeated founder events associated with rapid postglacial migrations, mixture of lineages originating from different glacial refugia, and possible interspecific gene flow make the genetic structure of these frontier populations highly dynamic. At the same time, the trailing populations at the southern margin of the species’ range often face decline when they are outcompeted by species better adapted to the new warmer climates (Hampe and Petit, 2005; Beckage et al., 2008) or cannot survive for other reasons regardless of competition. The projected rates of future climate change exceed those observed after the LGM (IPCC, 2022). For many Eastern North American tree species, the ecological optima will move northward at least by 100 km, and for several species the expected range shifts will likely exceed 250 km by the end of this century (Iverson and Prasad, 1998). Knowledge of the evolutionary history of a species is important to better understand its current state, making predictions of its fate in the future (Johnson et al., 2016; Johnson et al., 2018), and development of conservation programs if needed (Hoban et al., 2022) – particularly in the light of today’s rates of global climate change and human caused habitat fragmentation. This requires comprehensive analysis of genetic processes in tree populations at various evolutionary stages (Johnson et al., 2018) and spatial scales (LaRue et al., 2021) – from the expanding front to the declining trailing edge.

Red spruce (*Picea rubens* Sarg.) is an excellent model system to consider postglacial migration and evolution of northern temperate forest trees in relation to climate change and interspecific hybridization. because: (1) its northern populations are only a few generations old, whereas its southern populations are fragmented and currently restricted to high elevations, including potential refugia, and northern populations are genetically differentiated from southern populations; (2) it is sensitive to climate and environmental changes and has experienced massive diebacks from environmental change caused by the combined effects of climate warming, historical land use, and industrial pollution; and (3) it hybridizes naturally with black spruce (*Picea mariana* (Mill.) B.S.P.) in the northern parts of its range, but its evolutionary relationships with black spruce are not clear; and (4) very little is known about its postglacial migration and evolution.

Red spruce is an important and characteristic component of late-successional forests of eastern Canada and the northeastern United States. The natural range of red spruce covers territories from North Carolina in the United States to eastern Ontario and Nova Scotia in Canada (Burns and Honkala, 1990). In the northern part of its range, red spruce is one of the main components of cold temperate forests, whereas the southern populations are largely scattered and discontinuous, and occur either at mountainous sites, or in cool and moist wetlands (Burns and Honkala, 1990). Red spruce is characterized by relatively low overall genetic diversity (Eckert, 1989; Hawley and DeHayes, 1994; Perron et al., 2000; Rajora et al., 2000a) and a narrow ecological niche that make it sensitive to climate and environmental changes (DeHayes et al., 1991). A major decrease in the size of red spruce populations from 1800 onward has been associated, in part, with climate warming (Hamburg and Cogbill, 1988), though it should be noted that there have been some recent observations of red spruce re-expansion (Rollins et al., 2010). Since the 1960’s, a significant decline of red spruce populations has also been observed at many high elevation sites along the Appalachian Mountain chain (DeHayes and Hawley, 1992). This decline has been associated with the complex damaging effects of industrial emissions - reduced growth, defoliation and poor cold tolerance commonly observed in the affected stands (Friedland et al., 1984; McLaughlin et al., 1987; DeHayes et al., 1991; McLaughlin et al., 1993). Within the southern part of its range, red spruce is restricted to colder high elevation locations. These isolated populations of red spruce represent a valuable portion of the species’ gene pool, which are likely to be highly sensitive to climate warming.

In the northern parts of its range, red spruce is sympatric with closely related black spruce, where introgressive hybridization occurs between these two species (Morgenstern and Farrar, 1964; Manley, 1972; Gordon, 1976). Since the criteria for identification of interspecific hybrids between *P. rubens* and *P. mariana* are not rigid, estimates of the degree of introgression vary from extensive to minor among studies (Morgenstern and Farrar, 1964; Manley, 1972; Gordon, 1976; Fowler et al., 1988; Bobola et al., 1996; Perron and Bousquet, 1997). Additional analysis is required to understand the evolutionary relationships between red spruce and black spruce, as well as ongoing processes in the hybrid zones.

The history of the postglacial migrations in red spruce probably had significant influence on the population structure in this species, and it should be considered when dissecting the more recent effects of air pollution, logging, and climate change and developing a conservation genetic strategy for its forest genetic resources in North America. However, very little is known on postglacial migration and evolution of red spruce. Based on higher allozyme allelic richness and heterozygosity observed in northern than southern red spruce populations, Hawley and DeHayes (1994) speculated that these two population groups originated from two separate glacial refugia: one in the southern Appalachian Mountains, and another situated in the coastal areas, which later became continental shelf to the east of the mid-Atlantic states. On the other hand, the genetic distance analysis in their study did not show separation between the northern and southern populations as would be expected if the populations originated from two glacial refugia. The higher genetic diversity in the northern red spruce populations may be a result of natural hybridization with black spruce instead of their origins from separate glacial refugia. Spruce was widespread in the continental United States during the glaciation: various *Picea* macrofossils and pollen grains occur from the ice margin to east-central Louisiana but are absent in the Atlantic coastal areas (Jackson and Overpeck, 2000; Jackson et al., 2000). Fossil records indicate that red spruce survived in the southern Appalachians during glaciation (Jackson et al., 2000) and do not support the existence of the second red spruce refugium during LGM. Red spruce existed in central Appalachians around 15,000 years BP (Watts, 1979; Lindbladh et al., 2003). Coastal refugia for spruce may have existed during the warmer mid-Holocene period to play a significant role at the later stages of recolonization process, but not during the LGM (Schauffler and Jacobson, 2002). Pollen stratigraphies indicated that *P. rubens* was not widely represented in the northern part of its current range until 1000-1500 years BP (Lindbladh et al., 2003).

In the present study, we investigated range-wide genetic diversity and population genetic structure of red spruce and reconstructed the history of its postglacial migrations using highly variable microsatellite markers of the nuclear genome. We evaluated two concurrent hypotheses: 1.) whether the existing red spruce populations have originated from a single LGM refugium, or 2.) there have been multiple refugia. Under the 1.) hypothesis of recolonization from a single LGM refugium, isolation by distance and reduced genetic diversity levels in the northern populations associated with the repeated founder events may be expected. 2.) Multiple refugia can be revealed by clear genetic differentiation among lineages based on multilocus genetic structure. The migration time frames can be established from the recently available fossil pollen stratigraphies. We also assessed the evolutionary relationships between red spruce and black spruce and addressed the question whether red spruce is genetically depauperate using Bayesian analysis, highly variable molecular markers, and robust sample sizes.

## Materials and methods

### Red spruce populations, field sampling and DNA isolation

**Figure 1:**
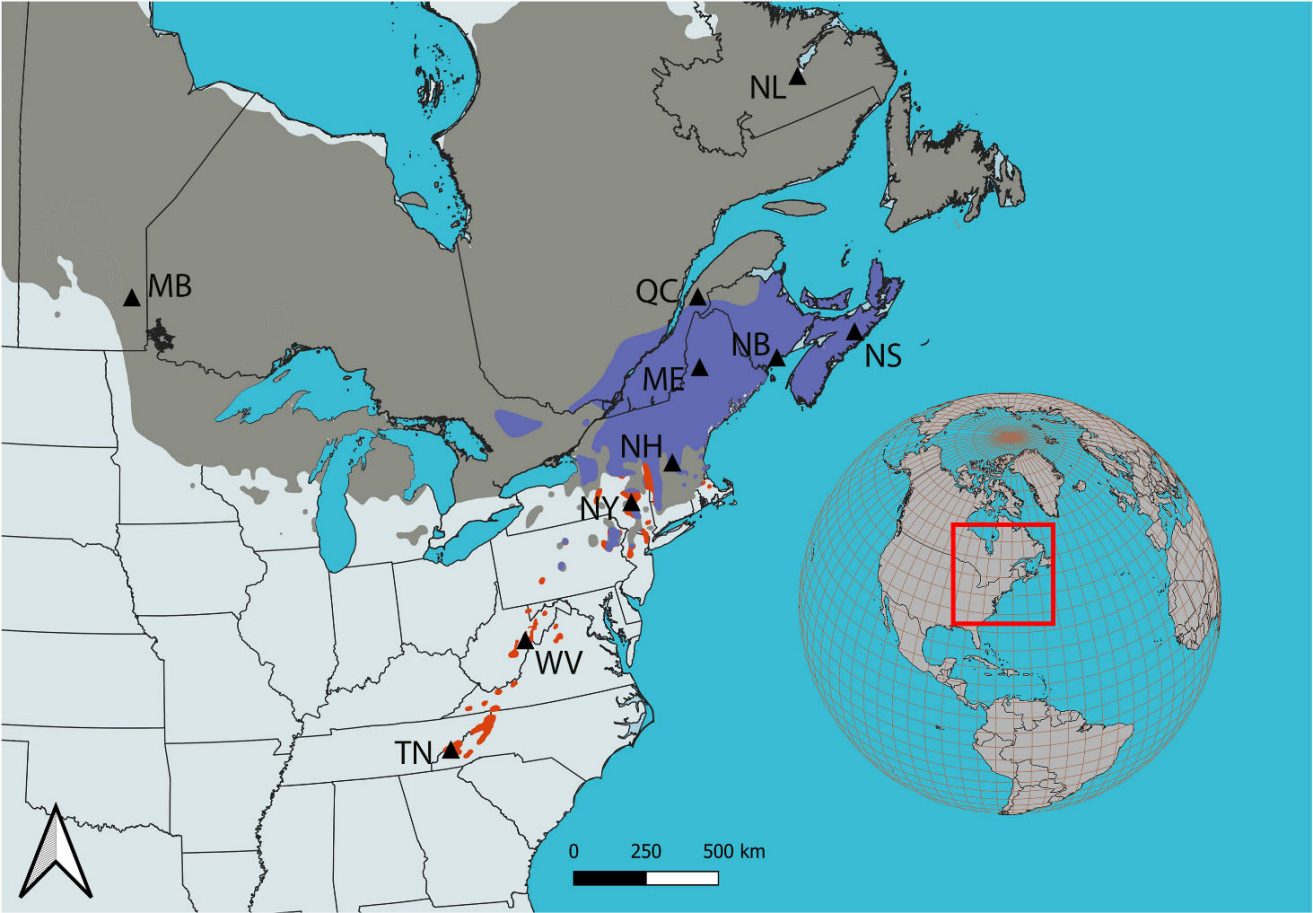
Geographic distribution of *Picea rubens* and *Picea mariana* in eastern North America, adapted from Little (1971). Species ranges are indicated by the red (southern red spruce), and dark grey (black spruce) shading. Purple shading indicates the northern red spruce sympatric zone with black spruce. Red box on the inset map indicates the extent of the study area. Sampled populations (back triangles) are indicated and labeled as per **Table 1**. Red spruce populations sampled: TN, Tennessee; WV, West Virginia; NH, New Hampshire; NY, New York; ME, Maine; QC, Quebec; NB, New Brunswick; NS, Nova Scotia; Black spruce: NL, Labrador; MB, Manitoba.

Eight populations of red spruce were sampled across the range of this species (**Figure 1**). Six populations formed a transect along the Appalachian Mountain chain, and two populations were from New Brunswick (NB) and Nova Scotia (NS), completing a latitudinal cross-section of the northern part of the species’ range. Five populations (WV, TN, NY, NB, and NS) were old-growth red spruce stands without significant human intervention documented in the past, and three (ME, QC, and NH) were represented by bulked seeds collected between 1997 and 2001 from about 50 mature trees (**Table 1**). Seeds were obtained from the Canadian Forest Service seed bank. Sixty mature trees, spaced at least 50 m apart to eliminate possible family structure effects, were sampled in the five old-growth red spruce populations, and sixty seeds were randomly selected from each of the three bulked-seed populations. Minimum spacing between trees was calculated based on the average seed dispersal distance (Burns and Honkala, 1990). To make sure that the bulked seed lots were not subject to a possible bottleneck effect, we compared their genetic diversity parameters with populations represented by the foliage samples. We found no significant difference in allelic richness and heterozygosity between the two sample groups. Possible heterozygosity fluctuations among seeds and adult trees due to the selection against highly inbred individuals should be negligible as the latter are mostly eliminated at the seed formation stage (O'Connell et al., 2006a). Using seed collections along with other sample types is a common practice when the natural populations cannot be accessed due to logistical reasons or harvesting (Hawley and DeHayes, 1994; Jaramillo-Correa et al., 2004).

**Table 1:** Red spruce and black spruce populations sampled and their geographic locations.

Population ID	Region	Location	Geographic coordinates		Elevation (m)
			Latitude	Longitude	
Red spruce populations					
TN	SRS	Mt. Clingmans Dome, Great Smoky Mountains National Park, Tennessee	35˚35’N	083˚28’W	800 m
WV	SRS	Gaudineer Knob, Monongahela National Forest, West Virginia	38˚38’N	079˚50’W	1242 m
NY	SRS	Mt. Rusk, Catskill Forest Preserve, New York	42˚12’ N	074˚16’W	1100 m
NH	NRS	Andorra Forest, New Hampshire	43˚05’N	072˚07’W	360 m
ME	NRS	Pittston Farm, Maine	45˚45’N	069˚45’W	n/a
QC	NRS	Lac Moreau, Quebec	47˚54’N	068˚51’W	330 m
NB	NRS	Loch Alva Lake, New Brunswick	45˚16’N	066˚18’W	150 m
NS	NRS	Abraham Lake, Nova Scotia	45˚10’N	062˚37’W	196 m
Black spruce populations					
MB	BS	Pine Falls, Manitoba	50˚41’N	095˚54’W	274 m
NL	BS	Goose Bay, Labrador	53˚17’N	060˚25’W	n/a

Red spruce populations were divided into southern (SRS) and northern (NRS) groups on the basis of their geographic location, UPGMA, and Bayesian genetic clustering.

The sample size of 60 individuals per population allowed us to capture most allelic diversity in red spruce populations, both in the evolutionary young northern and older southern populations (Bashalkhanov et al., 2009). A minimum sample size of 57 individuals was recommended to detect all alleles with the threshold frequency of 0.09 with a 95% probability (Gillet, 1999).

To estimate the possible effects of introgressive hybridization of *P. rubens* with *P. mariana* in the sympatric zone and to understand evolutionary relationships between these species, two pure black spruce populations were included in the analysis: Pine Falls population from Manitoba (MB) (Rajora and Pluhar, 2003), and Goose Bay population from Labrador (NL) (**Figure 1**; **Table 1**). The Pine Falls population sample consisted of needles from mature trees, whereas the Goose Bay population was represented by a bulked seed collection. These two populations of *P. mariana* possibly originated from separate glacial refugia, according to the mitochondrial haplotype distribution analysis (Jaramillo-Correa et al., 2004).

Foliage samples were collected in plastic bags with 10 g silica gel pouches as desiccant and kept at ambient temperatures in the field. Upon arrival to the lab, the samples were stored at -20˚C. Genomic DNA was isolated using a high-throughput magnetic fishing protocol (Bashalkhanov and Rajora, 2008).

### Microsatellite genotyping

Microsatellite markers targeting the biparentally-inherited nuclear (nu) genome were used to genotype the sampled individuals for various genetic diversity, population genetic structure, and phylogeography analyses.

Expressed Sequence Tag (EST)-based and genomic sequence-based microsatellite markers, previously developed in the Rajora lab for *P. glauca* and *P. mariana* (Shi et al., 2014; Rajora and Mann, 2021), were tested for amplification and polymorphism detection in red spruce. Genomic microsatellites had a higher proportion of null alleles. Only one showed the absence of null alleles during preliminary screening and was selected for future analysis. Most EST-based markers yielded good amplification results in *P. rubens*, *P. mariana*, and *P. glauca*. Eight EST-based microsatellites with 2-bp and 3-bp core repeats were selected for subsequent genotyping (**Table 2**). Mutation rates for 2-bp repeats are normally higher than that for 3-bp and 4-bp repeats (Chakraborty et al., 1997), and they may provide information about the evolutionary processes occurring at different time scales. One of the primers in each pair carried a standard M13 tail sequence to facilitate fluorescent labeling and detection. In total, genotypes of 600 trees were determined for nine microsatellite loci (**Table 2**; **Supplementary Data File 1**).

**Table 2:** Microsatellite primers sequences, amplification conditions, amplicon size range, and number of alleles observed in *Picea rubens*.

Locus ID^A^	Genebank Accession #	Primer sequences, 5’-3’	Amplification conditions		Repeat motif	Allele size range^1^	Number of alleles^2^
			T_A_, ˚C	MgCl_2_, mM			
*RPGSE03*	CN480906	F:M13*-AGCTAACTGGACTGGGACCTT R:CCGCACATGATATCCACAAG	50	1.5	(TTA)_6_	223-244	8
*RPGSE04*	CK442213	F:M13*-CTTGATTTTTGGCGATCGTT R:GAACCGGAGGAGATGGACTA	58	1.8	(CCG)_6_	200-248	16
*RPGSE05*	CK442392	F:M13**-CCGATTCAGGCAAGAGAATC R:TCACTGGCCACAGTTTATCG	50	1.5	(GAA)_6_	257-281	7
*RPGSE08*	CK443173	F:M13**-TCTCAAGAGAGGACGGAGGA R:GCATTCTGAGAGCCTTGCTT	50	1.5	(GAA)_6_	227-230	2
*RPGSE10*	CK441912	F:M13*-ATACGTTGGCGTTTCCGTCT R:TGAGGGCTTATGGACTACGC	59.5	1.5	(GGA)_6_	174-231	13
*RPGSE29*	CN480899	F:M13*-TGGCTTTTTATTCCAGCAAG R:GCCAGATTTTGCAAAGTGGA	52	1.5	(GA)_11_	241-287	23
*RPGSE34*	CN480905	F:M13*-CCAATTTGGTCCAATCTAGCA R:GGATGTGTTTTGGAGGGTTG	50	1.5	(GA)_10_	255-299	23
*RPGSE35*	CN480907	F:M13*-TGGCTCTCATCCAGAAAAGAA R:GGCTGCTCTCTTATCCGTTTT	52	1.5	(TA)_26_	151-207	25
*RPMSA13*	KJ847213	F:M13*-AACCATGAAACCCTAGCGACT R:TGAGGACTTAGGCCCACATT	53	1.8	(GA)_9_	171-295	25

*^A^RPGSE03, RPGSE04, RPGSE05, RPGSE08, RPGSE10, RPGSE29, RPGSE34*, and *RPGSE35* are genic microsatellite markers developed from *Picea glauca* EST sequences (Rajora and Mann, 2021). *RPMSA13* is a genomic microsatellite marker developed from AFLP fragment in *Picea mariana* (Shi et al., 2014).

*M13F-tail: 5’-IRDye700/800-CACGACGTTGTAAAACGAC-3’;

**M13R-tail: 5’-IRDye700/800-GGATAACAATTTCACACAGG-3’.

^1^ – Amplicon size, ^2^ – Total number of alleles in all populations studied.

Amplification reactions were performed in 10 μl reaction volume with 10 ng template DNA, 0.2 mM dNTP, 1.5-1.8 mM MgCl_2_, 0.25-0.83 pmol of each primer, 0.5 pmol of fluorescent labeled M13 primer (5’-IRDye700/800), 1x GoTaq Flexi Clear reaction buffer and 0.25 units of GoTaq Flexi DNA Polymerase (Promega, Madison, WI; Cat # M8295). Thermal cycling profiles were as follows: 2 min at 94°C, then 5 cycle touchdown step: 30 s at 94°C, 45 s initially at 65°C, then touchdown -2°C/cycle, 45 s at 72°C, followed by 33 cycles each of 30 s at 94°C, 45 s at the T_A_ (**Table 2**), 45 s at 72°C, and the final extension at 72°C for 5 min. The heating and cooling rates in the Eppendorf EP-S thermal cyclers were set to 6°C/s and 4.5°C/s, respectively.

Amplification products with incorporated fluorescent labels were separated on LiCor 4300 Genetic Analyzers (LiCor, Lincoln, NE). Up to 4 primer pairs were used in multiplex PCR with two loci running in each of the 700 nm and 800 nm IRDye detection channels. A minimum gap of 40 bp was maintained between loci to avoid overlapping of alleles. The gels were scored with SAGA GT/MX 3.3. software, followed by manual data verification. Alleles were designated based on their amplicon sizes.

### Genetic diversity analysis

Raw data were exported to a Microsoft Excel file. Data format conversions were performed with the Microsatellite Toolkit for Microsoft Excel (Park, 2001). Data quality for microsatellites was verified by the MICROCHECKER program (Van Oosterhout et al., 2004). MICROCHECKER can indicate possible presence of null alleles if there is an overall significant excess of homozygotes, and if it is evenly distributed across the homozygote classes. Then basic genetic diversity parameters, such as the mean number of alleles per locus, allele frequencies, expected and observed heterozygosity values, and the inbreeding coefficient (*F*_IS_) – were calculated using the Microsatellite Toolkit and R (R Core Team, 2022). Alleles specific to *P. rubens* and *P. mariana* were counted. The effective number of alleles per locus (A*_E_*) was determined as an inverse of expected homozygosity as described in (O'Connell et al., 2006b). Latent genetic potential (LGP) and genotypic additivity (richness) (G_A_) were calculated as described in Rajora et al. (2000b). Latent genetic potential (Bergmann et al., 1990) provides estimates for the content of rare and low frequency alleles in a population that might contribute to its adaptive potential under the changing environmental conditions. Genotypic additivity (Rajora et al., 2000b) describes the genotypic diversity in a population. Observed G_A_ is the total number of genotypes observed in a population summed over all the loci. The expected G_A_ is a sum of the theoretical number of single-locus genotypes in a diploid population. The Shannon’s information index (I) was calculated for each population. It has the advantage that it does not assume a population to be in the Hardy-Weinberg equilibrium, which may be unlikely in evolutionary young, or highly fragmented populations. One-way ANOVA was used for each genetic diversity measure to test the differences between the allopatric *P. rubens* and *P. mariana* populations, and the red spruce populations from the sympatric zone. Potential deviations from the Hardy-Weinberg equilibrium were assessed by Fisher’s exact test (Guo and Thompson, 1992) implemented in ARLEQUIN 3.5 (Excoffier and Lischer, 2010). The Ewens-Watterson neutrality test (Ewens, 1972; Watterson, 1978) was also carried out using ARLEQUIN 3.5 to examine the possible deviations in the observed genetic variation from the neutral expectations. Tests for possible bottleneck events and isolation by distance were carried out using BOTTLENECK 1.2.02 (Piry et al., 1999), and IBD 1.52 (Bohonak, 2002) programs, respectively. Effective population sizes were estimated through *θ* using the maximum likelihood coalescent approach implemented in the MIGRATE 3.0 program (Beerli, 2008), assuming the infinite allele mutation model for all markers, and the average mutation rate of 1x10^-3^, which is typical for microsatellite loci (Schlötterer and Wiehe, 1999; Marriage et al., 2009) and has been used in conifers (Pandey and Rajora, 2012; Rajora and Zinck, 2021). General statistical tests were carried out using the R statistical environment (R Core Team, 2022).

### Population genetic structure analysis

Genetic differentiation between populations was assessed by *G*_ST_ (Nei, 1973), *F*_ST_ (Weir and Cockerham, 1984), and *R*_ST_ (Slatkin, 1995), calculated using the FSTAT 2.9.3 program (Goudet, 2001). Several different approaches were used to determine the gene pool subdivision within red spruce and between red and black spruce. Groupwise *F*_ST_ and Nei’s genetic distance (Nei, 1972) estimates were determined using the R statistical program (R Core Team, 2022) and the ADEgenet package (Jombart, 2008). Population group comparisons were made based on various pairwise distance measures, including Nei’s genetic distance (Nei, 1972), *F*_ST_ (Weir and Cockerham, 1984), and Nm (Wright, 1931). The subdivision between the population groups was further tested by AMOVA using the ARLEQUIN 3.5 program. Bayesian clustering analysis implemented in STRUCTURE 2.3.4 (Pritchard et al., 2000) was used to infer the population structure with (location prior) and without referring to predefined geographical populations. Tests were run for K=1–14, in 10 iterations of 500,000 sweeps, plus 100,000 burn-in sweeps using the admixture and correlated allele frequency models. The number of populations/genetic groups in the data set was estimated using the ΔK parameter suggested by Evanno et al. (2005). Plots of population membership were constructed using CLUMPAK (Kopelman et al., 2015).

### Phylogenetic and phylogeography analyses

To infer the patterns of the postglacial expansions of red spruce, pairwise Nei’s genetic distances (Nei, 1972), *F*_ST_, and δμ^2^ (Goldstein et al., 1995) were calculated using the POPULATIONS 1.2.30 program (Langella, 1999) with 1,000 bootstrap replications. *F*_ST_ and standard genetic distances assume the infinite allele mutation model (IAM), and δμ^2^ is based on the stepwise mutation model (SMM). Unweighted pair group method with arithmetic mean (UPGMA) trees were constructed, and a majority rule consensus tree was produced. UPGMA is not based on any particular evolutionary model, which may be beneficial when the population differentiation is driven by a complex interplay of several factors. Maximum likelihood trees were also constructed with CONTML program from the PHYLIP 3.67 software package (Felsenstein, 2004).

### Population evolutionary history and admixture analysis

**Figure 2:**
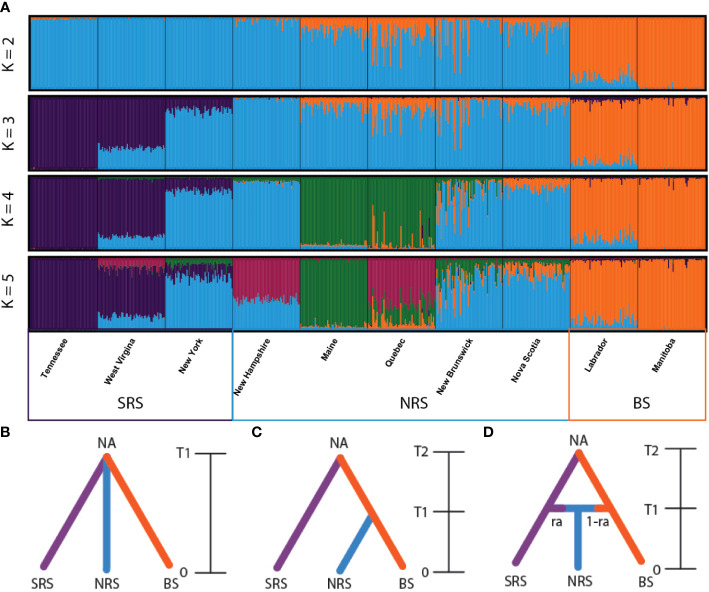
**(A)** Bar plot estimation of the membership coefficient (Q) for each spruce individual grouped on the geographic location. The figure is shown for K = 2 through 5, under the admixture model. The Evanno method (Evanno et al., 2005) suggested K=2 as the optimal clustering level (**Figure S2**). At K = 2 the split is between red and black spruce. K = 3 was selected as the sub-structure grouping level based on the Evano method (**Figure S3**). Red spruce populations: Tennessee, West Virginia, New York, New Hampshire, Maine, Quebec, New Brunswick, Nova Scotia. Black spruce: Labrador, Manitoba. Allopatric southern red spruce populations from Tennessee, West Virginia, and New York are well defined, while the northern populations show high degree of admixture. Note the presence of genotypes similar to red spruce lineages in “pure” black spruce population from Labrador. Historical patterns of admixture and introgression between red and black spruce were assessed using approximate Bayesian computation. Tested divergence scenarios included: **(B)** an admixture model where red and black spruce diverged from a common ancestor and the admixture population resulted from geneflow between the red and black spruce populations following divergence, **(C)** an ancestral model where all three populations diverged from a common ancestor, and **(D)** a hierarchical model where black and red spruce diverged from a common ancestor. The three populations tested include Southern Red Spruce (SRS) – blue line, Northern Red Spruce (NRS) – red line, and Black Spruce (BS) – green line. **(D)** NA = starting effective population size, T2 = divergence time (in generations) from most recent common ancestor, T1 = time (in generations) of admixture event, ra = gene flow rate from red spruce, and 1-ra is gene flow rate from black spruce. Relative posterior probabilities were computed to provide statistical support to select the most likely scenario. 1,000,000 simulations were performed, and the most likely scenario was evaluated by comparing the posterior probabilities using logistic regression on 1% of simulated datasets closest to the observed data.

To assess historical patterns and dynamics of admixture and introgression between red and black spruce approximate Bayesian computation was run using DIYABC 2.1.0 (Cornuet et al., 2014). First, a model of divergence from a common ancestor between red spruce and black spruce was compared to the alternative scenarios where either red spruce or black spruce split from one another. Following the finding that the most likely scenario was that the two species split from a common ancestor three possible divergence scenarios were tested: 1.) an ancestral model where all three populations diverged from a common ancestor (**Figure 2B**), 2.) a hierarchical model where black and red spruce diverged from a common ancestor and the admixed population diverged from black spruce (**Figure 2C**), and 3.) an admixture model where red and black spruce diverged from a common ancestor and the admixture population resulted from geneflow between the red and black spruce populations following divergence (**Figure 2D**). Red and black spruce populations were grouped based on their geographic location and Structure analysis, representing southern red spruce (TN, WV, NY), black spruce (NL and MB), and sympatric northern red spruce (NH, ME, QC, NB, NS). Relative posterior probabilities were computed to provide statistical support to select the most likely scenario. 1,000,000 simulations were performed, and the most likely scenario was evaluated by comparing the posterior probabilities using logistic regression on 1% of simulated datasets closest to the observed data. Following selection of the most likely scenario, parameters were estimated including effective population size and number of generations since divergence and admixture. We used 29 years as the selected generation time based on Capblancq et al. (2020) to extrapolate to number of years since divergence. Scenarios were assessed using principal components analysis comparing deviations of the summary statistics of the posterior predictive distribution to the observed data. In addition to the pooled population samples, we selected six groupings of individual populations to test individually. The groupings included all three southern individual red spruce populations (NY, WV, TN), one admixed population (NB), and MB or NL black spruce populations (**Supplementary Figure S1**). We estimated relative posterior probabilities and assessed their fit as outlined above. Following model selection, we estimated the scenario parameters.

## Results

### Allele composition and genetic diversity

Nuclear microsatellites with different core repeat sizes demonstrated contrasting patterns of molecular variation. Microsatellite markers with dinucleotide repeats had higher genetic variability (A=22.5) than the markers with trinucleotide repeats (A=7.8). This is consistent with the previously documented lower mutation rates in trinucleotide repeats lower than dinucleotide repeats (Chakraborty et al., 1997).

Overall, 139 alleles were detected at the nine nuclear SSR loci (**Table S1**). Out of those, 36 alleles were specific to red spruce, and 10 were specific to black spruce (**Table S1**). Allopatric populations of *P. rubens* and *P. mariana* had different distribution of allele frequencies (**Table S1**). For example, at the locus *RPGSE03*, allele 229 was the most common (frequency 0.66–1.00) in red spruce, whereas in black spruce it showed low frequency (0.09–0.11). On the other hand, alleles 232 and 238 at *RPGSE03* were common in black spruce but were absent in the southern allopatric red spruce populations and occurred at lower frequencies in the sympatric red spruce populations. The allele 198 at *RPGSE10* was common in black spruce (frequency 0.37–0.48), but rare in southern red spruce. Overall, frequencies of 16 alleles demonstrated significant correlation with latitude in red spruce populations (**Table S1**): 7 out of 39 alleles in microsatellite loci with 3-bp core repeat units, and 9 out of 90 alleles in microsatellites with 2-bp repeats. A highly significant positive correlation between the pairwise *F*_ST_ values and geographic distances among the 8 red spruce populations was observed for trinucleotide nuclear repeats (Mantel test *r*=0.74, *p*=0.001). This may be related to the increasing proportion of the black spruce alleles in the northern populations of *P. rubens*, rather than true isolation by distance. Once the loci *RPGSE03* and *RPGSE10* were removed from the analysis, the correlation between *F*_ST_ and geographic distance was no longer significant. Dinucleotide microsatellites had high numbers of low frequency alleles having limited effect on the observed *F*_ST_ values, thus they did not show significant isolation by distance in red spruce.

Observed (H_O_) and expected (H_E_) heterozygosity in red spruce populations varied between 0.36–0.44 and 0.49–0.62, respectively (**Table 3**). Moderate to significant heterozygote deficiency was detected in all populations, with average *F*_IS_=0.256, which is significantly higher than that previously reported for allozyme markers in red spruce (Hawley and DeHayes, 1994; Rajora et al., 2000a). Populations from Maine, Quebec, and New Brunswick had *F*_IS_ of 0.318, 0.340, and 0.299, respectively (**Table 3**). Wilcoxon’s rank test under the infinite allele mutation model (IAM) indicated an excess of heterozygotes in populations from New Hampshire and Quebec. However, with the limited number of loci employed, heterozygosity was a poor estimator of genome-wide inbreeding (Balloux et al., 2004). Significant deviations from the Hardy-Weinberg equilibrium were observed in all populations of red spruce, and they were more pronounced in the northern part of the species’ range (**Table S2**). Ewens-Watterson neutrality test did not reveal any significant evidence for selection (lowest *p*-value ~0.78).

**Table 3:** Population genetic diversity parameters in 8 red and 2 black spruce populations.

Population	A_T_	A (SE)	A_E_	H_E_ (SD)	H_O_ (SD)	*F*_IS_	LGP	G_A_(O)	G_A_(E)	I	*N_e_*
TN	63	7.00 (1.67)	1.95	0.487 (0.129)	0.437 (0.022)	0.104	58.35	127	352	1.120	364
WV	64	7.11 (1.78)	2.06	0.515 (0.132)	0.381 (0.021)	0.261	59.59	128	373	1.172	332
NY	60	6.67 (1.12)	2.00	0.500 (0.097)	0.376 (0.023)	0.251	55.45	96	275	1.050	326
NH	57	6.33 (1.39)	2.07	0.517 (0.113)	0.394 (0.021)	0.240	52.62	111	279	1.113	294
ME	69	7.67 (1.66)	2.27	0.560 (0.111)	0.383 (0.022)	0.318	64.99	129	398	1.261	346
QC	81	9.00 (1.91)	2.66	0.624 (0.103)	0.413 (0.021)	0.340	77.57	155	536	1.435	409
NB	69	7.67 (1.82)	2.07	0.517 (0.104)	0.363 (0.021)	0.299	64.61	117	418	1.166	340
NS	68	7.56 (1.56)	2.17	0.539 (0.104)	0.422 (0.022)	0.217	63.80	123	378	1.199	405
NL	85	9.44 (1.96)	2.45	0.591 (0.109)	0.406 (0.022)	0.316	81.27	161	581	1.440	394
MB	83	9.22 (1.96)	2.53	0.604 (0.103)	0.478 (0.022)	0.209	79.39	163	563	1.428	442
SRS	61A	6.81A	2.03A	0.506A	0.404A	0.202A	57A	122A	335A	1.135A	330A
NRS	69A	7.71A	2.23A	0.548A	0.391A	0.285A	65A	124A	401A	1.222A	365A
*p*_SRS-NRS_	0.140	0.138	0.235	0.208	0.536	0.125	0.141	0.886	0.307	0.348	0.248
RS	66B	7.38B	2.16A	0.532A	0.396A	0.254A	62B	123B	376B	1.190B	352A
BS	84A	9.33A	2.49A	0.597A	0.442A	0.263A	80A	162A	572A	1.434A	418A
*p*_RS-BS_	**0.012**	**0.012**	0.087	0.076	0.088	0.884	**0.013**	**0.015**	**0.013**	**0.023**	0.063

A_T_, total number of alleles for all loci; A, mean number of alleles; A_E_, effective number of alleles per locus; LGP, latent genetic potential; G_A_(O), observed number of genotypes (genotypic additivity); G_A_(E), expected genotypic additivity; I, Shannon’s information index; *Ne*, effective population size inferred from coalescent θ estimates. Population groups: SRS, Southern populations of *Picea rubens* (according to the Structure Analysis): TN, WV, and NY; NRS, Northern populations of *Picea rubens*: NH, ME, QC, NB, and NS; RS, All populations of red spruce; BS, All populations of *Picea mariana*: NL, and MB. Individual population names are listed in **Table 1**. p-values are given for one-way ANOVA among the corresponding population groups. Bold p values are significant at 95% confidence. Duncan means separation test at α=0.05 was done on the same groups.

Observed and expected heterozygosity and inbreeding coefficients were similar among *P. rubens* and *P. mariana*, but allelic richness, latent genetic potential, genotypic additivity, and Shannon diversity indices were significantly higher in black spruce. The northern red spruce populations had higher allelic and genotypic genetic diversity and *N_e_* (**Table 3**). However, the differences in genetic diversity levels and *N_e_* were not statistically significant between the northern and southern red spruce (**Table 3**). The two populations from NY and NH had the lowest latent genetic potential values.

### Population genetic structure and phylogeography

Microsatellite markers indicated moderate levels of genetic differentiation between the red spruce populations: total multilocus *F*_ST_ 8.5%, *R*_ST_ 6.6% for all microsatellite loci, and *F*_ST_ 10.0%, *R*_ST_ 8.5% for dinucleotide repeats, and *F*_ST_ 5.8%, *R*_ST_ 4.9% for trinucleotide repeats loci. Earlier studies based on allozyme markers reported *G*_ST_ of 7.5% (Hawley and DeHayes, 1994), and *F*_ST_ of 4.7% (Rajora et al., 2000a).

Bayesian clustering carried out using STRUCTURE 2.3.4. (Pritchard et al., 2000) and assessed using the Evano method (Evanno et al., 2005) suggested 2 major clusters based on the admixture model using both location prior (**Figure 2A**, **Figure S2**) and no location prior (**Figure S3**; **Figure S4**) models. Two minor peaks were also suggested at K=3 and K=4 (**Figures S2**, **S3**) indicating genetic substructure within the sampled populations. At K=2, the split is between red and black spruce, as expected (**Figure 2A**, **Figure S4**), and K=3 agrees with partitioning of populations between SRS, NRS, and BS (**Figure 2A**). The pure red spruce populations TN, WV and NY were consistently closely related, with both WV and NY sharing closer ancestry with TN. Five populations showed some level of increased admixture from BS including NH, ME, QC, NB, and NS. Pure *P. mariana* from NL and MB also were in good agreement. The “pure” black spruce from NL had some admixed red spruce lineages.

**Figure 3:**
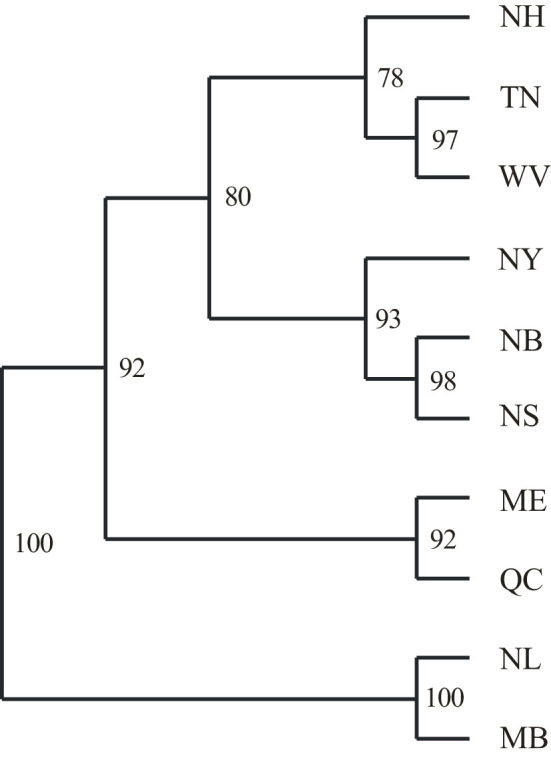
An unweighted pair group method with arithmetic mean (UPGMA) cluster plot of 8 red spruce and 2 black spruce populations based on Nei (1972) genetic distance for microsatellite markers. Population names and locations are provided in **Table 1**. Bootstrap support values are given for 1000 replications. Red spruce populations: TN – Tennessee, WV, West Virginia; NH, New Hampshire; NY , New York,; ME, Maine; QC, Quebec; NB, New Brunswick; NS, Nova Scotia. Black spruce: NL, Labrador; MB, Manitoba.

The UPGMA tree based on the Nei’s standard genetic distances (IAM model implied) was generally consistent with the geographic distribution of the sampled populations and formed four clusters: southern populations from TN and WV, along with NH; northern NB and NS, plus NY; ME and QC; and the two pure black spruce populations of NL and MB (**Figure 3**). SMM-based methods demonstrated poor resolution and absence of concordance with spatial distances. Northern red spruce populations share a significant proportion of black spruce alleles (**Table S1**) as a possible result of previously documented introgressive hybridization (Perron and Bousquet, 1997), which leads to biased *R*_ST_ and δμ^2^ estimates. Maximum likelihood trees generally confirmed the UPGMA clustering pattern, although bootstrap support among red spruce populations was weak (**Figure S5**). The Maine and Quebec populations were found to be genetically closer to *P. mariana*. Both populations are located in the middle of the introgression zone (Perron and Bousquet, 1997), and a higher proportion of black spruce alleles may be expected. Heterozygote deficiency in those two populations is probably related to the distribution of allele frequencies in hybrid populations leading to elevated H_E_, rather than actual inbreeding or sampling bias as the differences in H_O_ are not statistically significant from other populations. An old-growth red spruce population from New Brunswick has similar excess of H_E_. Groupwise AMOVA indicated that 3.40% of the genetic variation was between the northern and southern red spruce population groups, 3.47% among populations within groups and 93.13% within populations. This partition of the genetic variation was statistically significant (*P*=0.000 to 0.015). Pairwise genetic sub-division estimates indicate noticeable differentiation among the northern and southern populations of red spruce. At the same time, genetic differentiation between red and black spruce is significantly higher than the within-species subdivision (**Table 4**).

**Table 4:** Pairwise genetic subdivision estimates between and within population groups.

Groups	Nei GD	*F*_ST_	Nm
SRS–NRS	0.127 (0.084-0.213)	0.038	6.381
SRS–BS	0.381 (0.277-0.469)	0.171	1.210
NRS–BS	0.351 (0.224-0.585)	0.124	1.771
NRS–NRS	0.142 (0.037-0.289)	0.089	2.559
SRS–SRS	0.064 (0.045-0.083)	0.048	4.958
RS–RS	0.126 (0.037-0.289)	0.085	2.691
RS–BS	0.362 (0.224-0.585)	0.135	1.596
BS–BS	0.159	0.081	2.836

Nei GD– pairwise Nei (1972) genetic distances and range within and between population groups of *Picea rubens* and *Picea mariana*. *F*_ST_ – groupwise *F*_ST_ estimates. Nm – gene flow (Nm=(1/*F*_ST_-1)/4) (Wright, 1931). Population groups: SRS – Southern populations of *Picea rubens* TN, WV, and NY; NRS – Northern populations of *Picea rubens*: NH, ME, QC, NB, and NS; RS – All populations of red spruce; BS – All populations of *Picea mariana*: NL, and MB.

Approximate Bayesian computation identified the admixture model as the most likely scenario based on its higher posterior probability (0.8863) compared to the hierarchical split (0.0353) and divergence from a common ancestor scenario (0.0784) (**Figure S6**). Model checking indicated that the admixture scenario fits well with the data as indicated by the observed data centering on the cluster of posterior predictive distribution in the PCA (**Figure S7**). Values of effective population size based on the estimates of posterior distributions of parameters suggest that red spruce and black spruce expanded from an ancestral population to their current populations 4290 generations ago. An admixture event occurred around 343 generations ago (**Table 5**). At an assumed generation time of 29 years (Capblancq et al., 2020) the admixture event occurred following the LGM approximately 9,947 ybp. The admixture rates of both red and black spruce with the admixed population are 0.66 and 0.34 respectively. DIYABC does not consider geneflow following divergence, rather only admixture. Because there is pronounced substructure (based on Structure analysis) between SRS, NRS, and BS and genetic differentiation we are reasonably confident in our ability to assess our scenarios using approximate Bayesian computation. Assessment of individual populations resulted in similar results, with the admixture scenario identified as the most likely model among the six population groupings and the admixture event occurring between 218-1890 generations ago depending on the population combinations compared (**Table S5**; **Figure S1**).

**Table 5:** Parameter estimates from approximate Bayesian computation admixture scenario.

	Parameter	Mean	Median	Mode
SRS	*N_e_1*	4440.00	4220.00	3360.00
BS	*N_e_2*	5750.00	5670.00	5470.00
NRS	*N_e_3*	6960.00	7150.00	7930.00
Admix	*t1*	343.00	246.00	186.00
RS Admixture Rate	*Ra*	0.66	0.69	0.66
BS Admixture Rate	*1-ra*	0.34	0.31	0.34
MRCA	*t2*	4290.00	3860.00	3050.00
	*NA*	1800.00	1030.00	88.30

N_e_1, effective population size of SRS; N_e_2, effective population size of BS; N_e_3, effective population size of NRS. NA, starting effective population size; t2, divergence time (in generations) from most recent common ancestor; t1, time (in generations) of admixture event; ra, gene flow rate from red spruce; and 1-ra is the gene flow rate from black spruce. Generation time is assumed to be 29 years. MRCA, most recent common ancestor.

## Discussion

### Genetic diversity of red spruce

Our results suggest that red spruce has moderate levels of genetic variation. It may be difficult to compare genetic diversity estimates obtained from microsatellite with that obtained from allozyme markers due to the inherently different mutation rates (Avise, 2004). Nevertheless, microsatellite genetic diversity levels were about 4-5 times higher than that of allozyme genetic diversity levels reported in red spruce (**Table 6**). Microsatellite allelic diversity observed in red spruce populations (A=6.3–9.0, average 7.4) was like that documented in Sitka spruce-*Picea sitchensis* (A=5.7–10.0, average 7.7 (Mimura and Aitken, 2007), and sympatric white spruce, *Picea glauca* (average A=6.83) based on EST microsatellites (Fageria and Rajora, 2013) but lower than observed in this species based on genomic (A=16.38; Rajora et al., 2005) or genomic and EST microsatellites (average A=11.38; Fageria and Rajora, 2013) (**Table 6**) and *P. mariana* (A=9.22-9.44; this study – **Table 3**). There is variation in mutation rates among different microsatellite loci resulting in different levels of genetic variation revealed by these loci, which makes study-to-study comparisons difficult. In our study, genetic diversity estimates for *P. rubens* and *P. mariana* populations were obtained using the same set of microsatellite markers. Our study suggests that red spruce has lower allelic diversity, latent genetic potential, expected and observed genotypic richness than black spruce for the same microsatellite markers (**Table 3**). However, the genetic diversity observed in red spruce in our study was similar to that reported for transcontinental white spruce based on six EST microsatellites (Fageria and Rajora, 2013), three of which were the same as we used in our study. Because eight of the nine microsatellite markers we used in our study were EST microsatellites, a comparison with the results obtained from EST microsatellites is more valid than from genomic microsatellites as genetic diversity at EST microsatellites is much lower than at genomic microsatellites (Fageria and Rajora, 2013; Fageria and Rajora, 2014; Rajora et al., 2023).

**Table 6:** Genetic diversity and fixation index (*F*) estimates in representative *Picea* species with endemic or regional (R) and widespread or transcontinental (T) distribution.

Species	Distribution	A	H_O_	H_E_	*F*	Marker system	Reference
*P. rubens*	R	1.60	0.097	0.100	0.030	Allozymes	(Rajora et al., 2000a)
*P. rubens*	R	1.6	0.091	–	–	Allozymes	(Eckert, 1989)
*P. rubens*	R	2.4	0.078	–	–	Allozymes	(McLaughlin et al., 1993)
*P. rubens*	R	1.47	0.075	0.079	0.051	Allozymes	(Hawley and DeHayes, 1994)
*P. martinezii*	R	1.39	0.104	0.111	0.063	Allozymes	(Ledig et al., 2000)
*P. omorika*	R	1.50	0.073	0.067	-0.090	Allozymes	(Ballian et al., 2006)
*P. obovata*	T	2.91	0.161	0.168	0.042	Allozymes	(Kravchenko et al., 2008)
*P. mariana*	T	2.52	0.222	0.308	–	Allozymes	(Rajora and Pluhar, 2003)
*P. mariana*	T	2.70	0.339	0.300	-0.130	Allozymes	(Isabel et al., 1995)
*P. glauca*	T	3.03	0.342	0.344	0.006	Allozymes	(O'Connell et al., 2006b)
*P. rubens*	R	1.60	0.069	0.077	–	c-DNA STS	(Perron et al., 2000)
*P. mariana*	T	2.00	0.103	0.122	–	c-DNA STS	(Perron et al., 2000)
*P. mariana*	T	7.6	0.430	0.590		SSR (EST)	(Rajora and Mann, 2021)
*P. mariana*	T	9.38	0.401	0.609	–	SSR (genomic)	(Shi et al., 2014)
*P. rubens*	R	6.20	0.381	0.618	–	SSR (genomic)	(Shi et al., 2014)
*P. glauca*	T	16.38	0.649	0.851	0.237	SSR (genomic)	(Rajora et al., 2005)
*P. glauca*	T	11.38	0.529	0.655	0.177	SSR (EST+genomic)	(Fageria and Rajora, 2013)
*P.glauca*	T	6.83	0.412	0.488	0.147	SSR (EST)	(Fageria and Rajora, 2013)
*P. glauca*	T	10.57	0.490	0.637	0.210	SSR (EST+ genomic)	(Fageria and Rajora, 2014)
*P. glauca*	T	9.18	0.470	0.648	0.292	SSR (EST+genomic)	(Rajora et al., 2023)
*P. abies*	T	15.14	0.494	0.634	0.221	SSR	(Meloni et al., 2007)
*P. sitchensis*	R	7.70	–	0.678	–	SSR (genomic)	(Mimura and Aitken, 2007)
*P. mariana*	T	9.33	0.442	0.597	0.260	SSR	Present study
*P. rubens*	R	7.38	0.396	0.532	0.256	SSR	Present study
P. rubens*	R	–	0.008	0.094	–	Exome SNP	(Capblancq et al., 2021)

- =not reported.

A, Number of alleles per locus; H_O_, observed heterozygosity; H_E_, expected heterozygosity.

* = H_0_ and H_E_ means calculated from Capblancq et al., 2021 **Supplemental Data**.

Species with narrower or regional distribution ranges typically have lower genetic diversity than the species with broad or transcontinental ranges (Hamrick et al., 1992). This is well reflected in *Picea rubens* and *Picea sitchensis*, which have similar geographic distribution patterns following the dominant mountain systems in their respective regions. Measured with the same marker system (allozymes or microsatellites), allelic diversity and heterozygosity in red spruce were generally lower than in transcontinental or wide-spread spruce species (e.g., *P. glauca*, *P. mariana, P. abies*), but similar to other spruce species that have regional or narrow distribution ranges or to transcontinental white spruce based on EST microsatellites (**Table 6**). Previously reported allelic diversity for allozyme markers in red spruce varied from A=1.47 (Hawley and DeHayes, 1994) to A=1.60 (Rajora et al., 2000a) and 2.40 (Eckert, 1989). This is similar to the mean allozyme allelic diversity of A=1.83 alleles per locus averaged across 102 studies in gymnosperms (Hamrick et al., 1992). Although *P. rubens* has lower overall genetic diversity levels than sympatric black spruce and white spruce and some other spruce species with very wide or transcontinental distributions, we cannot conclude that this species is genetically depauperate as previously reported (Perron et al., 2000).

Southern populations of red spruce demonstrated slightly lower nuclear microsatellite allelic (11.6%) and genotypic (1.6% or 16.5%) diversity, *N_e_* (9.6%) and expected heterozygosity (7.7%) compared to the northern populations, although the differences were not statistically significant (**Table 3**). Hawley and DeHayes (1994) also reported 4.5% higher allelic richness in the northern red spruce populations. Introgressive hybridization with *P. mariana* may have enriched the allelic diversity in the northern populations of *P. rubens* in the sympatric zone. On balance, southern red spruce populations showed slightly higher (3.3%) observed heterozygosity than the northern populations. This is in contrast with the report of higher observed heterozygosity (0.0885) in northern versus southern (0.0747) populations by Hawley and DeHayes (1994).

Our results demonstrated that red spruce and black spruce have a substantial number of species-specific alleles for the nuclear microsatellites, and that the northern red spruce populations have significant influx of black spruce alleles (**Table S1**). Deviations from the Hardy-Weinberg equilibrium were observed in all populations of red spruce (**Table S2**). Repeated colonization events and introgressive hybridization with *P. mariana* in the northern part of the species range, and migration to uplands and air pollution effects in the southern populations may be the likely cause of these deviations (Bashalkhanov et al., 2013).

Our study suggests that most of the nuclear genetic diversity resides among individuals within populations in red spruce with inter-population genetic differentiation of about 8% (**Tables 4**). These results are consistent with those obtained previously for red spruce with allozymes (Hawley and DeHayes, 1994; Rajora et al., 2000a) and are typical for conifers. The results indicate that the southern red spruce populations are more genetically differentiated from black spruce than the northern populations (**Tables 4**). This may be a result of more recent introgressive hybridization with black spruce in the sympatric northern populations. Our data also demonstrate that the southern populations of red spruce are less genetically differentiated (*F*_ST_=0.048) and have higher gene flow (Nm=4.96) among themselves than the northern populations (*F*_ST_=0.089; Nm=2.56). Indeed, the lowest *F*_ST_ and highest gene flow was observed between TN and WV populations (*F*_ST_=0.033; Nm=7.33) (**Table S4**). In contrast, Hawley and DeHayes (1994) reported higher allozyme divergence among southern versus northern red spruce populations. In our study, the two southernmost red spruce populations from Tennessee and West Virginia show a high degree of genetic similarity despite having been in isolation since mid-Holocene, which indicates that genetic drift has not played a significant role so far in these populations. Microsatellite data suggest significant gene flow occurred between these populations in the past: Nm=7.33, which contradicts previously reported high divergence between the southern red spruce populations (Nm=1.6) and the subsequent assumption of manifestation of genetic drift in southern populations (Hawley and DeHayes, 1994). The southern TN and WV populations may be remnants of a pure red spruce glacial refugium in Appalachians documented earlier by fossil data (Jackson et al., 2000).

### Postglacial migrations and evolution of *Picea rubens*, and *Picea rubens* – *Picea mariana* complex

Lack of pronounced isolation by distance among the red spruce populations across the current distribution range, found in our study, may be an indicator of a somewhat complex post-glacial migration history of this species. Late Quaternary pollen and macrofossil data are available for many sites worldwide. In conjunction with other records of climate and environmental change they provide valuable information about the distribution of extant and extinct taxa at various spatial and temporal scales. Spruce macrofossils during the Last Glacial Maximum (ca 21000 years BP) were found at most sites to the south of the ice margin, and they are most abundant in Mississippi and Louisiana (Jackson et al., 2000). Molecular phylogeographic reconstruction indicated that the Mississippi Valley may have been one of the several glacial refugia for *P. mariana* (Jaramillo-Correa et al., 2004). With the retreat of the ice shield, spruce was among the first species to colonize the newly exposed areas – a rapid migration northeast was recorded approximately 16000-12000 years BP, followed by a recession during warmer mid-Holocene periods (Watts, 1979; Jackson et al., 2000; Lindbladh et al., 2003). Cooler areas on the East Coast may have served as a secondary refugium during that period (Jackson and Overpeck, 2000; Schauffler and Jacobson, 2002; Lindbladh et al., 2003). Fossil pollen records indicate that red spruce was rare in New England during late Glacial and early Holocene (14000-8000 years BP), except for two smaller sites in the coastal area (Lindbladh et al., 2003), and it probably coexisted there with black spruce during the Late Holocene (last 1400 years), at least in Maine. It did not exist in the northeastern part of its current range until 1000-500 years BP (Lindbladh et al., 2003). It is often challenging to separate *P. rubens* and *P. mariana* from their pollen alone, as they have a great deal of similarity in morphology (Lindbladh et al., 2003). This means that *P. rubens* may be underestimated in the palaeoecological record where it was rare and there are some challenges in assessing the pollen record of *P. rubes* due to this scarcity.

As the red spruce populations from TN and WV are genetically close, and given the previously published fossil data, we suggest that during the Last Glacial Maximum red spruce probably occupied a single refugium in Southern Appalachians, and then migrated northward along the East Coast. Being a late successional, shade-tolerant species, it was much less abundant than the early successional black spruce, which perhaps migrated to the New England states from its Mississippi refugium. During the recession in warmer mid-Holocene, the cooler and moist Atlantic coastal areas may have sheltered both *P. rubens* and *P. mariana*. Since the two species are closely related, extensive introgressive hybridization may have occurred. Cooler climates in the region observed during the last 1500 years facilitated another rapid range expansion of red spruce and black spruce. The contemporary sympatric populations of red and black spruce may be descendants from the secondary mid-Holocene refugium, whereas southern allopatric red spruce populations probably represent the remnants of its glacial refugium.

Results from approximate Bayesian computation suggest a pattern of admixture with introgression occurring between red spruce and black spruce beginning approximately 343 generations ago. Beginning 4290 generations ago with an ancestral *N_e_* of 1800, red spruce and black spruce diverged from their common ancestor. Then, around 343 generations ago, following the LGM, recontact and introgression occurred in the sympatric zone. This scenario explains the current genetic subdivision of red spruce into northern and southern variants indicated by the maximum likelihood and IAM-based trees and Bayesian structure clustering, as well as the influx of black spruce-specific alleles in the northern red spruce populations, which is detectable even in the old-growth stands. Earlier the presence of red spruce-specific mitotype in the eastern populations of black spruce was reported (Jaramillo-Correa et al., 2004). Occurrence of red spruce mitotype in the pure populations of *P. mariana* in the sympatric zone is an important indication of the two-way nature of the hybridization process. Furthermore, significant interspecific gene flow was documented in morphologically “pure” sympatric populations of *P. rubens* and *P. mariana* with species-specific RAPD markers (Nkongolo et al., 2003). Although RAPD markers have certain inherent limitations (dominance, questionable band homology, inconsistency), their results are in good agreement with the microsatellite data in the present study. We found a significant proportion of black spruce alleles in the presumably pure old-growth red spruce stands from Abraham Lake, Nova Scotia, and Loch Alva Lake, New Brunswick. Inversely, Bayesian approach suggested existence of admixed red spruce lineages in the pure black spruce population from Goose Bay, Labrador. Genetic distances based on allozyme data published by Hawley and DeHayes (1994), also provide evidence of hybridization between red spruce and black spruce in New Brunswick and Nova Scotia, although the authors did not consider it significant. Since the reproductive barriers between *P. rubens* and *P. mariana* are weak, but still significant (Major et al., 2005), these findings can be interpreted as a result of long-term introgression, rather than local interspecific gene flow, which is consistent with the post-glacial migration and hybridization scenario outlined above. Previous studies of the introgressive hybridization between *P. rubens* and *P. mariana* may have underestimated the scale of the process.

### Red spruce – black spruce evolutionary relationships

Based on high genetic similarity between red spruce and black spruce and the observation that genetic diversity in red spruce was a subset of the genetic diversity in black spruce, it has been hypothesized that *P. rubens* is a derivative species from *P*. *mariana* (Perron et al., 2000; Jaramillo-Correa and Bousquet, 2003). However, this conclusion is questionable because species-specific polymorphisms for red spruce and black spruce have been identified in both nuclear and organellar genomes (Bobola et al., 1992; Perron et al., 1995; Bobola et al., 1996; Germano and Klein, 1999; Nkongolo et al., 2003), and fossil evidence suggests that these species have co-existed in various locations since the LGM (Watts, 1979; Jackson et al., 2000; Lindbladh et al., 2003).

Although black spruce shares a significant proportion of its allelic diversity with red spruce, both species were found to have very distinct patterns of distribution of microsatellite alleles. Thirty-six microsatellite alleles were specific to red spruce and ten microsatellite alleles were specific to black spruce (**Table S1**). In a previous study, a number of species-specific impenetrable SNP loci were identified in *P. rubens* and *P. mariana* (de Lafontaine et al., 2015). This makes it difficult to conclude that the overall genetic diversity of *P. rubens* is a subset of *P. mariana*’s gene pool suggesting that the previously reported progenitor-derivative relationship between these two species (Perron et al., 2000; Jaramillo-Correa and Bousquet, 2003) is unlikely. This conclusion is further supported by the co-existence of these species in the fossil records (Watts, 1979; Jackson et al., 2000; Lindbladh et al., 2003), higher karyotype similarities of black spruce to white spruce than to red spruce (Nkongolo, 1996), and presence of many species-specific genetic variants in both the nuclear and organellar genomes (Bobola et al., 1992; Perron et al., 1995; Bobola et al., 1996; Germano and Klein, 1999; Nkongolo et al., 2003). A broader phylogenetic reconstruction of the *Picea* genus identified *P. rubens* as a sister species, rather than a derivative of *P. mariana* (Lockwood et al., 2013; Parmar et al., 2022), which further corroborates our viewpoint. Recent isolation and genetic drift were hypothesized to be the main driving forces for red spruce speciation (Perron et al., 2000; Jaramillo-Correa and Bousquet, 2003). However, we did not find any significant evidence for bottleneck events and genetic drift in the southern pure red spruce populations. Our results, based on sufficiently large population sample sizes (60), are in contrast from results using genomic markers that have shown a reduction in population size and long-term population decline in *N_e_* (potentially indicating drift) following the LGM based on whole exome sequencing (Capblancq et al., 2020). However, the Capblancq et al. (2020) study was based on a very small sample size of 2 to 11 individuals per population. While the two species are closely related and obviously share a common ancestor, the existence of shared alleles may be a result of common ancestry, relatively recent gene flow, and introgression occurring after the LGM as indicated by our approximate Bayesian analysis. A similar picture was reported in the interspecific hybridization zones for *Quercus* in Europe (Lexer et al., 2006) and *Larix* in Asia (Semerikov and Lascoux, 2003).

We, therefore, conclude that red spruce is not likely a derivative species from black spruce and the postglacial migration history and introgressive hybridization should be considered when describing evolutionary relationships between these two closely related species.

### Conservation implications

Previous large-scale climatic fluctuations have played a major role in shaping the current population structure of red spruce, causing long distance migrations and gene pool rearrangement. Given the projected climate warming rates, we expect that this species might face a significant evolutionary impact in the near future. The isolated pure red spruce populations in Southern Appalachians have maintained their genetic diversity levels and they represent a significant proportion of the species’ gene pool. Although these populations have little commercial value, they form an essential habitat for several endangered plant and animal species (Blum, 1990). Warming climates may eliminate the ecological niche for the high elevation southern red spruce populations within the next several decades. The central populations of red spruce in New Hampshire and Vermont may become more fragmented because of the rapid elevation shift in their ecological optima (Beckage et al., 2008), which places them under the risk of extinction in the future. Introgressive hybridization with *P. mariana* enriches the genetic diversity in *P. rubens*, while these two species remain ecologically different. Conservation efforts to preserve the ecologically important pure red spruce in Southern Appalachians should be strengthened. Considerable advances have been achieved in developing genomic resources and molecular markers for *Picea*, and future population genomics studies should take advantage of this newly developed resources, while taking into account the minimum sample sizes required for making reliable inference based on observed genetic variation.

## Data availability statement

The original contributions presented in the study are included in the article/**Supplementary Material**, further inquiries can be directed to the corresponding author.

## Author contributions

OR: Conceptualization, Funding acquisition, Investigation, Methodology, Project administration, Resources, Supervision, Writing – original draft, Writing – review & editing. SB: Conceptualization, Formal Analysis, Investigation, Methodology, Writing – original draft, Writing – review & editing. JJ: Formal Analysis, Writing – original draft, Writing – review & editing.

## References

[B1] Avise J. C. (2004). Molecular markers, natural history, and evolution (London, UK: Chapman and Hall).

[B2] Ballian D. Longauer R. Mikić T. Paule L. Kajba D. Gömöry D. (2006). Genetic structure of a rare European conifer, Serbian spruce (Picea omorika (Panč.) Purk.). Plant Systematics Evol. 260 (1), 53–63. doi: 10.1007/s00606-006-0431-z

[B3] Balloux F. Amos W. Coulson T. (2004). Does heterozygosity estimate inbreeding in real populations? Mol. Ecol. 13 (10), 3021–3031. doi: 10.1111/j.1365-294X.2004.02318.x 15367117

[B4] Bashalkhanov S. Eckert A. J. Rajora O. P. (2013). Genetic signatures of natural selection in response to air pollution in red spruce (*Picea rubens, Pinaceae*). Mol. Ecol. 22 (23), 5877–5889. doi: 10.1111/mec.12546 24118331

[B5] Bashalkhanov S. Pandey M. Rajora O. P. (2009). A simple method for estimating genetic diversity in large populations from finite sample sizes. BMC Genet. 10 (1), 84. doi: 10.1186/1471-2156-10-84 20003542 PMC2800116

[B6] Bashalkhanov S. Rajora O. P. (2008). Protocol: A high-throughput DNA extraction system suitable for conifers. Plant Methods 4, 1–6. doi: 10.1111/1755-0998.12387 18673554 PMC2518145

[B7] Beckage B. Osborne B. Gavin D. G. Pucko C. Siccama T. Perkins T. (2008). A rapid upward shift of a forest ecotone during 40 years of warming in the Green Mountains of Vermont. Proc. Natl. Acad. Sci. 105 (11), 4197–4202. doi: 10.1073/pnas.0708921105 18334647 PMC2393766

[B8] Beerli P. (2008) Migrate version 3.0 - a maximum likelihood and Bayesian estimator of gene flow using the coalescent. Available at: http://popgen.scs.edu/migrate.html.

[B9] Bergmann F. Gregorius H. R. Larsen J. B. (1990). Levels of genetic variation in European silver fir (*Abies alba*). Genetica 82 (1), 1–10. doi: 10.1007/BF00057667

[B10] Blum B. M. (1990). *Picea rubens* Sarg. - Red spruce. Agric. Handbook US Department Agric. 1, 250–259.

[B11] Bobola M. S. Eckert R. T. Klein A. S. (1992). Restriction fragment variation in the nuclear ribosomal DNA repeat unit within and between *Picea rubens* and *Picea mariana* . Can. J. For. Res. 22 (2), 255–263. doi: 10.1139/x92-033

[B12] Bobola M. S. Guenette D. Eckert R. T. Klein A. S. Stapelfeldt K. Smith D. E. (1996). Using nuclear and organelle DNA markers to discriminate among *Picea rubens Picea mariana*, and their hybrids. Can. J. For. Res. 26 (3), 433–443. doi: 10.1139/x26-049

[B13] Bohonak A. J. (2002). IBD (Isolation by Distance): A program for analyses of Isolation by Distance. J. Heredity 93 (2), 153–154. doi: 10.1093/jhered/93.2.153 12140277

[B14] Burns R. M. Honkala B. H. (1990). “"Silvics of North America, conifers. Agriculture handbook 654,” in Tech rep. (Washington DC: USDA).

[B15] Capblancq T. Butnor J. R. Deyoung S. Thibault E. Munson H. Nelson D. M.. (2020). Whole-exome sequencing reveals a long-term decline in effective population size of red spruce (*Picea rubens*). Evolutionary Appl. 13 (9), 2190–2205. doi: 10.1111/eva.12985 PMC7513712 33005218

[B16] Capblancq T. Munson H. Butnor J. R. Keller S. R. (2021). Genomic drivers of early-life fitness in Picea rubens. Conserv. Genet. 22 (6), 963–976. doi: 10.1007/s10592-021-01378-7

[B17] Chakraborty R. Kimmel M. Stivers D. N. Davison L. J. Deka R. (1997). Relative mutation rates at di-, tri-, and tetranucleotide microsatellite loci. Proc. Natl. Acad. Sci. 94 (3), 1041–1046. doi: 10.1073/pnas.94.3.1041 9023379 PMC19636

[B18] Cornuet J.-M. Pudlo P. Veyssier J. Dehne-Garcia A. Gautier M. Leblois R.. (2014). DIYABC v2.0: a software to make approximate Bayesian computation inferences about population history using single nucleotide polymorphism, DNA sequence and microsatellite data. Bioinformatics 30 (8), 1187–1189. doi: 10.1093/bioinformatics/btt763 24389659

[B19] DeHayes D. H. Hawley G. J. (1992). Genetic implications in the decline of red spruce. Water Air Soil pollut. 62 (3), 233–248. doi: 10.1007/BF00480258

[B20] DeHayes D. H. Thornton F. C. Waite C. E. Ingle M. A. (1991). Ambient cloud deposition reduces cold tolerance of red spruce seedlings. Can. J. For. Res. 21 (8), 1292–1295. doi: 10.1139/x91-180

[B21] de Lafontaine G. Prunier J. Gérardi S. Bousquet J. (2015). Tracking the progression of speciation: variable patterns of introgression across the genome provide insights on the species delimitation between progenitor–derivative spruces (Picea mariana × P. rubens). Mol. Ecol. 24 (20), 5229–5247. doi: 10.1111/mec.13377 26346701

[B22] Eckert R. T. (1989). “Genetic variation in red spruce and its relation to forest decline in northeastern United States,” in Air pollution and forest decline. (Proc. 14th Intl. Meeting Specialists Air Pollution Effects in Forest Ecosystems. Eds. Bucher J. B. Bucher-Wallin I. (Birmensodorf: IUFRO P2.05).

[B23] Evanno G. Regnaut S. Goudet J. (2005). Detecting the number of clusters of individuals using the software STRUCTURE: a simulation study. Mol. Ecol. 14 (8), 2611–2620. doi: 10.1111/j.1365-294X.2005.02553.x 15969739

[B24] Ewens W. J. (1972). The sampling theory of selectively neutral alleles. Theor. Population Biol. 3 (1), 87–112. doi: 10.1016/0040-5809(72)90035-4 4667078

[B25] Excoffier L. Lischer H. E. L. (2010). Arlequin suite ver 3.5: a new series of programs to perform population genetics analyses under Linux and Windows. Mol. Ecol. Resour. 10 (3), 564–567. doi: 10.1111/j.1755-0998.2010.02847.x 21565059

[B26] Fageria M. S. Rajora O. P. (2013). Effects of harvesting of increasing intensities on genetic diversity and population structure of white spruce. Evolutionary Appl. 6 (5), 778–794. doi: 10.1111/eva.12064 PMC5779121 29387165

[B27] Fageria M. S. Rajora O. P. (2014). Effects of silvicultural practices on genetic diversity and population structure of white spruce in Saskatchewan. Tree Genet. Genomes 10 (2), 287–296. doi: 10.1007/s11295-013-0682-0

[B28] Felsenstein J. (2004). PHYLIP (Phylogeny Inference Package) version 3.6 (University of Washington, Seattle: Department of Genome Sciences).

[B29] Fowler D. P. Park Y. S. Gordon A. G. (1988). Genetic variation of red spruce in the Maritimes. Can. J. For. Res. 18 (6), 703–709. doi: 10.1139/x88-107

[B30] Friedland A. J. Gregory R. A. Karenlampi L. Johnson A. H. (1984). Winter damage to foliage as a factor in red spruce decline. Can. J. For. Res. 14 (6), 963–965. doi: 10.1139/x84-173

[B31] Germano J. Klein A. S. (1999). Species-specific nuclear and chloroplast single nucleotide polymorphisms to distinguish *Picea glauca*, *P. mariana* and *P. rubens* . Theor. Appl. Genet. 99 (1), 37–49. doi: 10.1007/s001220051206

[B32] Gillet E. M. (1999). “Minimum sample sizes for sampling genetic marker distributions,” in Final Compendium of the Research Project "Development, optimization and validation of molecular tools for assessment of biodiversity in forest trees" in the European Union DGXII Biotechnology FW IV Research Programme Molecular Tools for Biodiversity. Ed. Gillet E. M.

[B33] Goldstein D. B. Linares A. R. Cavallie-Sforza L. L. Feldman M. W. (1995). An evaluation of genetic distance for use with microsatellite loci. Genetics 139, 463–471. doi: 10.1093/genetics/139.1.463 7705647 PMC1206344

[B34] Gordon A. G. (1976). The taxonomy and genetics of *Picea rubens* and its relationship to *Picea mariana* . Can. J. Bot. 54 (9), 781–813. doi: 10.1139/b76-084

[B35] Goudet J. (2001). FSTAT, a program to estimate and test gene diversities and fixation indices (version 2.9.3).

[B36] Guo S. W. Thompson E. A. (1992). Performing the exact tes of Hary-Weinberg proportion for multiple alleles. Biometrics 48, 361–372. doi: 10.2307/2532296 1637966

[B37] Hamburg S. P. Cogbill C. V. (1988). Historical decline of red spruce populations and climatic warming. Nature 331 (6155), 428–431. doi: 10.1038/331428a0

[B38] Hampe A. Petit R. J. (2005). Conserving biodiversity under climate change: the rear edge matters. Ecol. Lett. 8 (5), 461–467. doi: 10.1111/j.1461-0248.2005.00739.x 21352449

[B39] Hamrick J. L. Godt M. J. W. Sherman-Broyles S. L. (1992). “Factors influencing levels of genetic diversity in woody plant species,” in Population Genetics of Forest Trees: Proceedings of the International Symposium on Population Genetics of Forest Trees Corvallis, Oregon, U.S.A., July 31–August 2,1990. Eds. Adams W. T. Strauss S. H. Copes D. L. Griffin A. R. (Dordrecht: Springer Netherlands), 95–124.

[B40] Hawley G. J. DeHayes D. H. (1994). Genetic diversity and population structure of red spruce (*Picea rubens*). Can. J. Bot. 72 (12), 1778–1786. doi: 10.1139/b94-219

[B41] Hoban S. Archer F. I. Bertola L. D. Bragg J. G. Breed M. F. Bruford M. W.. (2022). Global genetic diversity status and trends: towards a suite of Essential Biodiversity Variables (EBVs) for genetic composition. Biol. Rev. 97 (4), 1511–1538. doi: 10.1111/brv.12852 35415952 PMC9545166

[B42] IPCC (2022). Climate change 2022: Impacts, adaptations, and vulnerability. Contributions of working group II to the sixth assessment report of the intergovernmental panel on climage change (Geneva, Switzerland: Intergovernmental Panel on Climate Change).

[B43] Isabel N. Beaulieu J. Bousquet J. (1995). Complete congruence between gene diversity estimates derived from genotypic data at enzyme and random amplified polymorphic DNA loci in black spruce. Proc. Natl. Acad. Sci. 92 (14), 6369–6373. doi: 10.1073/pnas.92.14.6369 7603998 PMC41519

[B44] Iverson L. R. Prasad A. M. (1998). Predicting abundance of 80 tree species following climate change in the eastern United States. Ecol. Monogr. 68 (4), 465–485. doi: 10.1890/0012-9615(1998)068[0465:PAOTSF]2.0.CO;2

[B45] Jackson S. T. Overpeck J. T. (2000). Responses of plant populations and communities to environmental changes of the late Quaternary. Paleobiology 26 (sp4), 194–220. doi: 10.1666/0094-8373(2000)26[194:roppac]2.0.co;2

[B46] Jackson S. T. Webb R. S. Anderson K. H. Overpeck J. T. Webb Iii T. Williams J. W.. (2000). Vegetation and environment in Eastern North America during the Last Glacial Maximum. Quaternary Sci. Rev. 19 (6), 489–508. doi: 10.1016/S0277-3791(99)00093-1

[B47] Jaramillo-Correa J. P. Beaulieu J. Bousquet J. (2004). Variation in mitochondrial DNA reveals multiple distant glacial refugia in black spruce (*Picea mariana*), a transcontinental North American conifer. Mol. Ecol. 13 (9), 2735–2747. doi: 10.1111/j.1365-294X.2004.02258.x 15315685

[B48] Jaramillo-Correa J. P. Bousquet J. (2003). New evidence from mitochondrial DNA of a progenitor-derivative species relationship between black spruce and red spruce (*Pinaceae*). Am. J. Bot. 90 (12), 1801–1806. doi: 10.3732/ajb.90.12.1801 21653356

[B49] Johnson J. S. Gaddis K. D. Cairns D. M. Lafon C. W. Krutovsky K. V. (2016). Plant responses to global change: Next generation biogeography. Phys. Geogr. 37, 93–119. doi: 10.1080/02723646.2016.1162597

[B50] Johnson J. S. Krutovsky K. V. Rajora O. P. Gaddis K. D. Cairns D. M. (2019). “Advancing biogeography through population genomics,” in Population Genomics: Concepts, Approaches and Applications. Ed. Rajora O. P. (Springer:Nature Switzerland AG, Cham).

[B51] Jombart T. (2008). adegenet: a R package for the multivariate analysis of genetic markers. Bioinformatics 24, 1403–1405. doi: 10.1093/bioinformatics/btn129 18397895

[B52] Kopelman N. M. Mayzel J. Jakobsson M. Rosenberg N. A. Mayrose I. (2015). Clumpak: a program for identifying clustering modes and packaging population structure inferences across K. Mol. Ecol. Resour. 15, 1179–1191. doi: 10.1111/1755-0998.12387 25684545 PMC4534335

[B53] Kravchenko A. N. Larionova A. Y. Milyutin L. I. (2008). Genetic polymorphism of Siberian spruce (Picea obovata Ledeb.) in Middle Siberia. Russian J. Genet. 44 (1), 35–43. doi: 10.1134/S1022795408010055 18409386

[B54] Langella O. (1999) Populations. Available at: http://bioinformatics.org/~tryphon/populations/.

[B55] LaRue E. A. Rohr J. Knott J. Dodds W. K. Dahlin K. M. Thorp J. H.. (2021). The evolution of macrosystems biology. Front. Ecol. Environ. 19 (1), 11–19. doi: 10.1002/fee.2288

[B56] Ledig F. T. Bermejo-Velázquez B. Hodgskiss P. D. Johnson D. R. Flores-López C. Jacob-Cervantes V. (2000). The mating system and genic diversity in Martínez spruce, an extremely rare endemic of México's Sierra Madre Oriental: an example of facultative selfing and survival in interglacial refugia. Can. J. For. Res. 30 (7), 1156–1164. doi: 10.1139/x00-052

[B57] Lexer C. Kremer A. Petit R. J. (2006). COMMENT: Shared alleles in sympatric oaks: recurrent gene flow is a more parsimonious explanation than ancestral polymorphism. Mol. Ecol. 15 (7), 2007–2012. doi: 10.1111/j.1365-294X.2006.02896.x 16689915

[B58] Lindbladh M. Jacobson G. L. Schauffler M. (2003). The postglacial history of three *Picea* species in New England, USA. Quaternary Res. 59 (1), 61–69. doi: 10.1016/S0033-5894(02)00023-6

[B59] Lockwood J. D. Aleksić J. M. Zou J. Wang J. Liu J. Renner S. S. (2013). A new phylogeny for the genus Picea from plastid, mitochondrial, and nuclear sequences. Mol. Phylogenet. Evol. 69 (3), 717–727. doi: 10.1016/j.ympev.2013.07.004 23871916

[B60] Major J. E. Mosseler A. Johnsen K. H. Rajora O. P. Barsi D. C. Kim K. H.. (2005). Reproductive barriers and hybridity in two spruces, *Picea rubens* and *Picea mariana*, sympatric in eastern North America. Can. J. Bot. 83 (2), 163–175. doi: 10.1139/b04-161

[B61] Manley S. A. M. (1972). The occurrence of hybrid swarms of red and black spruces in central New Brunswick. Can. J. For. Res. 2 (4), 381–391. doi: 10.1139/x72-060

[B62] Marriage T. N. Hudman S. Mort M. E. Orive M. E. Shaw R. G. Kelly J. K. (2009). Direct estimation of the mutation rate at dinucleotide microsatellite loci in *Arabidopsis thaliana* (Brassicaceae). Heredity 103 (4), 310–317. doi: 10.1038/hdy.2009.67 19513093 PMC2749907

[B63] McLaughlin S. B. Downing D. J. Blasing T. J. Cook E. R. Adams H. S. (1987). An analysis of climate and competition as contributors to decline of red spruce in high elevation Appalachian forests of the Eastern United states. Oecologia 72 (4), 487–501. doi: 10.1007/BF00378973 28312509

[B64] McLaughlin S. B. Tjoelker M. G. Roy W. K. (1993). Acid deposition alters red spruce physiology: laboratory studies support field observations. Can. J. For. Res. 23 (3), 380–386. doi: 10.1139/x93-055

[B65] Meloni M. Perini D. Binelli G. (2007). The distribution of genetic variation in Norway spruce (Picea abies Karst.) populations in the western Alps. J. Biogeogr. 34 (6), 929–938. doi: 10.1111/j.1365-2699.2006.01668.x

[B66] Mimura M. Aitken S. N. (2007). Adaptive gradients and isolation-by-distance with postglacial migration in Picea sitchensis. Heredity 99 (2), 224–232. doi: 10.1038/sj.hdy.6800987 17487214

[B67] Morgenstern E. K. Farrar J. L. (1964). “Natural hybridization in red spruce and black spruce,” in Technical Report No. 4 (Toronto, Ontario, Canada: University of Toronto).

[B68] Nei M. (1972). Genetic distance between populations. Am. Nat. 106 (949), 283–&. doi: 10.1086/282771

[B69] Nei M. (1973). Analysis of gene diversity in subdivided populations. Proc. Natl. Acad. Sci. U. States America 70 (12), 3321–3323. doi: 10.1073/pnas.70.12.3321 PMC427228 4519626

[B70] Nkongolo K. K. (1996). Chromosome analysis and DNA homology in three *Picea* species, *P. mariana, P. rubens*, and *P. glauca* (*Pinaceae*). Plant Systematics Evol. 203 (1), 27–40. doi: 10.1007/BF00985235

[B71] Nkongolo K. K. Deverno L. Michael P. (2003). Genetic validation and characterization of RAPD markers differentiating black and red spruces: molecular certification of spruce trees and hybrids. Plant Systematics Evol. 236 (3), 151–163. doi: 10.1007/s00606-002-0236-7

[B72] O'Connell L. M. Mosseler A. Rajora O. P. (2006a). Impacts of forest fragmentation on the reproductive success of white spruce (*Picea glauca*). Can. J. Bot. 84 (6), 956–965. doi: 10.1139/b06-051 16912700

[B73] O'Connell L. M. Mosseler A. Rajora O. P. (2006b). Impacts of forest fragmentation on the mating system and genetic diversity of white spruce (*Picea glauca*) at the landscape level. Heredity 97 (6), 418–426. doi: 10.1038/sj.hdy.6800886 16912700

[B74] Pandey M. Rajora O. P. (2012). Genetic diversity and differentiation of core vs. peripheral populations of eastern white cedar, *Thuja occidentalis* (*Cupressaceae*). Am. J. Bot. 99 (4), 690–699. doi: 10.3732/ajb.1100116 22473976

[B75] Park S. D. E. (2001). Trypanotolerance in West African cattle and the population genetic effects of selection (University of Dublin).

[B76] Parmar R. Cattonaro F. Phillips C. Vassiliev S. Morgante M. Rajora O. P. (2022). Assembly and annotation of red spruce (Picea rubens) chloroplast genome, identification of simple sequence repeats, and phylogenetic analysis in Picea. Int. J. Mol. Sci. 23 (23), 15243. doi: 10.3390/ijms232315243 36499570 PMC9739956

[B77] Perron M. Bousquet J. (1997). Natural hybridization between black spruce and red spruce. Mol. Ecol. 6 (8), 725–734. doi: 10.1046/j.1365-294X.1997.00243.x

[B78] Perron M. Gordon A. G. Bousquet J. (1995). Species-specific RAPD fingerprints for the closely related *Picea mariana* and *P. rubens* . Theor. Appl. Genet. 91 (1), 142–149. doi: 10.1007/BF00220871 24169680

[B79] Perron M. Perry D. J. Andalo C. Bousquet J. (2000). Evidence from sequence-tagged-site markers of a recent progenitor-derivative species pair in conifers. Proc. Natl. Acad. Sci. 97 (21), 11331–11336. doi: 10.1073/pnas.200417097 11016967 PMC17200

[B80] Piry S. Luikart G. Cornuet J. M. (1999). Computer note. BOTTLENECK: a computer program for detecting recent reductions in the effective size using allele frequency data. J. Heredity 90 (4), 502–503. doi: 10.1093/jhered/90.4.502

[B81] Pritchard J. K. Stephens M. Donnelly P. (2000). Inference of population structure using multilocus genotype data. Genetics 155 (2), 945–959. doi: 10.1093/genetics/155.2.945 10835412 PMC1461096

[B82] Rajora O. P. Fageria M. S. Fitzsimmons M. (2023). Effects of Wild Forest Fires on Genetic Diversity and Population Structure of a Boreal Conifer, White Spruce (Picea glauca (Moench) Voss): Implications for Genetic Resource Management and Adaptive Potential under Climate Change. Forests 14 (1), 157. doi: 10.3390/f14010157

[B83] Rajora O. P. Mann I. K. (2021). Development and characterization of Novel EST-based single-copy genic microsatellite DNA markers in white spruce and black spruce. Mol. Biol. Rep. 48 (3), 2963–2971. doi: 10.1007/s11033-021-06231-1 33635471

[B84] Rajora O. P. Mann I. K. Shi Y.-Z. (2005). Genetic diversity and population structure of boreal white spruce (Picea glauca) in pristine conifer-dominated and mixedwood forest stands. Can. J. Bot. 83 (9), 1096–1105. doi: 10.1139/b05-083

[B85] Rajora O. P. Mosseler A. Major J. E. (2000a). Indicators of population viability in red spruce, *Picea rubens.* II. Genetic diversity, population structure, and mating behavior. Can. J. Bot. 78 (7), 941–956. doi: 10.1139/b00-066

[B86] Rajora O. P. Pluhar S. A. (2003). Genetic diversity impacts of forest fires, forest harvesting, and alternative reforestation practices in black spruce (*Picea mariana*). Theor. Appl. Genet. 106 (7), 1203–1212. doi: 10.1007/s00122-002-1169-9 12748771

[B87] Rajora O. P. Rahman M. H. Buchert G. P. Dancik B. P. (2000b). Microsatellite DNA analysis of genetic effects of harvesting in old-growth eastern white pine (*Pinus strobus*) in Ontario, Canada. Mol. Ecol. 9 (3), 339–348. doi: 10.1046/j.1365-294x.2000.00886.x 10736031

[B88] Rajora O. P. Zinck J. W. R. (2021). Genetic Diversity, Structure and Effective Population Size of Old-Growth vs. Second-Growth Populations of Keystone and Long-Lived Conifer, Eastern White Pine (Pinus strobus): Conservation Value and Climate Adaptation Potential. Front. Genet. 12. doi: 10.3389/fgene.2021.650299 PMC8388927 34456961

[B89] R Core Team (2022). R: A language and environment for statistical computing. R Foundation for Statistical Computing, (Vienna, Austria: R Foundation for Statistical Computing). URL https://www.R-project.org/

[B90] Rollins A. Adams H. Stephenson S. (2010). Changes in forest composition and structure across the red spruce-hardwood ecotone in the central Appalachians. Castanea 75 (3), 303–314. doi: 10.2179/09-052.1

[B91] Schauffler M. Jacobson J. G. L. (2002). Persistence of coastal spruce refugia during the Holocene in northern New England, USA, detected by stand-scale pollen stratigraphies. J. Ecol. 90 (2), 235–250. doi: 10.1046/j.1365-2745.2001.00656.x

[B92] Schlötterer C. Wiehe T. (1999). “Microsatellites, a neutral marker to infer selective sweeps,” in Microsatellites: evolution and applications. Ed. Goldsmith D. (New York: Oxford University Press).

[B93] Semerikov V. L. Lascoux M. (2003). Nuclear and cytoplasmic variation within and between Eurasian Larix (*Pinaceae*) species. Am. J. Bot. 90 (8), 1113–1123. doi: 10.3732/ajb.90.8.1113 21659211

[B94] Shi Y.-Z. Forneris N. Rajora O. P. (2014). Highly Informative Single-Copy Nuclear Microsatellite DNA Markers Developed Using an AFLP-SSR Approach in Black Spruce (Picea mariana) and Red Spruce (P. rubens). PloS One 9 (8), e103789. doi: 10.1371/journal.pone.0103789 25126846 PMC4134192

[B95] Slatkin M. (1995). A measure of population subdivision based on microsatellite allele frequencies. Genetics 139 (1), 457–462. doi: 10.1093/genetics/139.1.457 7705646 PMC1206343

[B96] Van Oosterhout C. Hutchinson W. F. Wills D. P. M. Shipley P. (2004). micro-checker: software for identifying and correcting genotyping errors in microsatellite data. Mol. Ecol. Notes 4 (3), 535–538. doi: 10.1111/j.1471-8286.2004.00684.x

[B97] Watterson G. A. (1978). The homozygosity test of neutrality. Genetics 88 (2), 405–417. doi: 10.1093/genetics/88.2.405 17248803 PMC1213809

[B98] Watts W. A. (1979). Late Quaternary vegetation of central Appalachia and the New Jersey coastal plain. Ecol. Monogr. 49 (4), 427–469. doi: 10.2307/1942471

[B99] Weir B. S. Cockerham C. C. (1984). Estimating F-statistics for the analysis of population structure. Evolution 38 (6), 1358–1370. doi: 10.2307/2408641 28563791

[B100] Wright S. (1931). Evolution in mendelian populations. Genetics 16 (2), 97–159. doi: 10.1093/genetics/16.2.97 17246615 PMC1201091

