# Supplementary material for: Postglacial phylogeography, admixture, and evolution of red spruce (Picea rubens Sarg.) in Eastern North America
Source: Front Plant Sci. 2023 Oct 12;14:1272362. doi: 10.3389/fpls.2023.1272362 (PMC10602686; doi:10.3389/fpls.2023.1272362)
Supplement: Supplementary file 1 [file DataSheet_1.docx]

**Supplementary Material**

Postglacial phylogeography, admixture, and evolution of red spruce

(*Picea rubens* Sarg.) in Eastern North America

Stanislav Bashalkhanov^1,2^, Jeremy S. Johnson^3^, Om P. Rajora^1*^

^1^ Faculty of Forestry and Environmental Management University of New Brunswick 28 Dineen Drive, Fredericton, NB, E3B 5A3, Canada

^2^ Current address: Maritime Provinces Higher Education Commission, 82 Westmorland Street, Suite 401, PO Box 6000, Fredericton, NB E3B 5 H1, Canada

^3^ Department of Forestry, Michigan State University, East Lansing, MI, 48824, USA

*** Correspondence:**Prof. Om P. Rajora

E-mail: [Om.Rajora@unb.ca](mailto:Om.Rajora@unb.ca)

Keywords: Postglacial migration, Glacial refugium, Genetic diversity and population structure, Interspecific hybridization, Molecular evolution, Approximate Bayesian Computation, Biogeography.

**Table S1.** Allele frequencies at individual microsatellite loci for all *Picea* populations.

| Locus | Populations | | | | | | | | | |
| --- | --- | --- | --- | --- | --- | --- | --- | --- | --- | --- |
| *RPGSE03* | TN | WV | NY | NH | ME | QC | NB | NS | NL | MB |
| 223 |  |  | 0.0196 |  | 0.0100 | 0.0172 |  |  | 0.0091 |  |
| 226 |  |  | 0.0098 |  |  |  |  |  | 0.0091 |  |
| 229* | 1.0000 | 1.0000 | 0.9216 | 0.8879 | 0.9200 | 0.6552 | 0.8091 | 0.8246 | 0.1091 | 0.0877 |
| 232* |  |  | 0.0196 | 0.1034 | 0.0700 | 0.2241 | 0.0364 | 0.1491 | 0.6091 | 0.5965 |
| 235R |  |  | 0.0196 |  |  |  | 0.0455 |  |  |  |
| 238 |  |  |  |  |  | 0.0690 | 0.1000 | 0.0263 | 0.1545 | 0.2368 |
| 241 |  |  |  | 0.0086 |  | 0.0172 |  |  | 0.0818 | 0.0351 |
| 244 |  |  | 0.0098 |  |  | 0.0172 | 0.0091 |  | 0.0273 | 0.0439 |
|  |  |  |  |  |  |  |  |  |  |  |
| *RPGSE04* | TN | WV | NY | NH | ME | QC | NB | NS | NL | MB |
| 200 |  |  |  |  | 0.0083 |  |  |  | 0.0603 | 0.0536 |
| 203 |  |  |  |  |  | 0.0167 |  |  | 0.0172 |  |
| 206B |  |  |  |  |  |  |  |  | 0.0086 | 0.0089 |
| 209B |  |  |  |  |  |  |  |  | 0.0086 |  |
| 215 |  | 0.0086 | 0.0119 |  |  |  |  |  |  | 0.0089 |
| 218* | 0.2018 | 0.0086 | 0.0476 |  |  |  | 0.0085 | 0.0254 | 0.0172 | 0.0268 |
| 221 | 0.0526 | 0.2155 | 0.2976 | 0.2667 | 0.3083 | 0.1167 | 0.2797 | 0.2119 | 0.0086 |  |
| 224* | 0.2193 | 0.0259 | 0.1071 |  | 0.0250 | 0.0250 | 0.0254 |  | 0.1810 | 0.2411 |
| 227 |  | 0.1724 | 0.0952 | 0.3000 | 0.1250 | 0.1750 | 0.0678 | 0.0847 | 0.0086 | 0.0893 |
| 230 | 0.2105 | 0.2414 | 0.1548 | 0.0167 | 0.1917 | 0.1333 | 0.1441 | 0.1271 | 0.4914 | 0.5000 |
| 233* | 0.0088 |  | 0.0476 | 0.1333 | 0.0250 | 0.1667 | 0.0593 | 0.1017 | 0.0862 | 0.0179 |
| 236 | 0.2895 | 0.3103 | 0.2143 | 0.1000 | 0.3083 | 0.2333 | 0.3559 | 0.4322 | 0.0948 | 0.0536 |
| 239R |  |  |  | 0.1250 | 0.0083 | 0.1083 | 0.0593 |  |  |  |
| 242 | 0.0088 |  |  | 0.0417 |  | 0.0083 |  | 0.0085 | 0.0172 |  |
| 245R | 0.0088 |  |  | 0.0083 |  |  |  |  |  |  |
| 248R |  | 0.0172 | 0.0238 | 0.0083 |  | 0.0167 |  | 0.0085 |  |  |
|  |  |  |  |  |  |  |  |  |  |  |
| *RPGSE05* | TN | WV | NY | NH | ME | QC | NB | NS | NL | MB |
| 257R |  |  |  |  |  | 0.0083 |  |  |  |  |
| 260 |  | 0.0083 |  |  |  |  |  | 0.0085 | 0.0088 |  |
| 263 | 0.0083 |  | 0.2895 | 0.0500 | 0.0917 | 0.1417 | 0.0667 | 0.0169 | 0.0526 | 0.0847 |
| 266 | 0.9917 | 0.9917 | 0.7105 | 0.9500 | 0.9083 | 0.8500 | 0.9250 | 0.9322 | 0.9035 | 0.8983 |
| 269 |  |  |  |  |  |  |  | 0.0424 | 0.0351 | 0.0169 |
| 281R |  |  |  |  |  |  | 0.0083 |  |  |  |
|  |  |  |  |  |  |  |  |  |  |  |
| *RPGSE08* | TN | WV | NY | NH | ME | QC | NB | NS | NL | MB |
| 227 | 1.0000 | 1.0000 | 0.9417 | 1.0000 | 0.9667 | 1.0000 | 1.0000 | 1.0000 | 1.0000 | 1.0000 |
| 230R |  |  | 0.0583 |  | 0.0333 |  |  |  |  |  |
|  |  |  |  |  |  |  |  |  |  |  |
| *RPGSE10* | TN | WV | NY | NH | ME | QC | NB | NS | NL | MB |
| 174 |  |  |  |  |  | 0.0085 |  |  | 0.0263 |  |
| 177R |  |  |  |  |  | 0.0169 |  |  |  |  |
| 180B |  |  |  |  |  |  |  |  |  | 0.0098 |
| 192* | 0.6339 | 0.5333 | 0.4186 | 0.4322 | 0.2895 | 0.3390 | 0.2414 | 0.4259 | 0.3333 | 0.3137 |
| 195* | 0.0446 |  | 0.0116 |  |  |  |  |  | 0.0965 |  |
| 198 | 0.0446 | 0.0750 | 0.0465 | 0.0678 | 0.1579 | 0.2034 | 0.3276 | 0.2963 | 0.3684 | 0.4804 |
| 201 | 0.2768 | 0.3833 | 0.5233 | 0.4915 | 0.5351 | 0.3983 | 0.4310 | 0.2593 | 0.1316 |  |
| 204 |  | 0.0083 |  |  |  |  |  |  |  | 0.0196 |
| 210 |  |  |  |  | 0.0175 | 0.0339 |  | 0.0185 | 0.0439 | 0.1078 |
| 213B |  |  |  |  |  |  |  |  |  | 0.0098 |
| 216B |  |  |  |  |  |  |  |  |  | 0.0392 |
| 219B |  |  |  |  |  |  |  |  |  | 0.0196 |
| 231R |  |  |  | 0.0085 |  |  |  |  |  |  |
|  |  |  |  |  |  |  |  |  |  |  |
| *RPGSE29* | TN | WV | NY | NH | ME | QC | NB | NS | NL | MB |
| 241B |  |  |  |  |  |  |  |  | 0.0125 |  |
| 243 |  |  |  |  | 0.0256 |  | 0.0192 | 0.0510 | 0.2375 |  |
| 245 |  |  |  |  | 0.0256 | 0.0351 |  |  | 0.0625 | 0.1186 |
| 247 |  |  |  | 0.0089 | 0.0385 |  | 0.0288 | 0.0510 | 0.3000 | 0.2627 |
| 249 |  |  |  | 0.0179 |  | 0.0965 |  |  | 0.1125 | 0.0678 |
| 251 |  |  |  | 0.0714 |  |  |  |  |  | 0.2458 |
| 253B |  |  |  |  |  |  |  |  | 0.0125 | 0.0339 |
| 255R |  | 0.0085 | 0.0109 |  | 0.0513 |  |  | 0.0204 |  |  |
| 257R |  | 0.0085 |  | 0.0089 |  | 0.0088 |  |  |  |  |
| 259R |  |  | 0.0109 | 0.0089 | 0.0513 | 0.0088 |  |  |  |  |
| 261R | 0.1091 | 0.0932 | 0.0326 |  | 0.2051 |  | 0.0769 | 0.0306 |  |  |
| 263 | 0.0455 | 0.1695 | 0.1522 | 0.1071 | 0.1026 | 0.0351 | 0.1346 | 0.1224 | 0.0250 |  |
| 265R | 0.0727 | 0.0424 | 0.0109 | 0.0804 | 0.0128 | 0.0789 |  | 0.0102 |  |  |
| 267 | 0.0091 | 0.0169 |  |  | 0.0513 | 0.0175 | 0.0385 | 0.0102 |  | 0.0085 |
| 269 | 0.0818 | 0.1356 | 0.0761 |  | 0.1026 | 0.0175 | 0.0769 | 0.0306 | 0.0125 | 0.0085 |
| 271 | 0.2818 | 0.2034 | 0.3804 | 0.2946 | 0.1923 | 0.0088 | 0.4327 | 0.3163 | 0.0750 | 0.0254 |
| 273 | 0.2545 | 0.1610 | 0.0652 | 0.1964 | 0.1154 | 0.2982 | 0.0865 | 0.1327 | 0.0625 | 0.0508 |
| 275 | 0.0909 | 0.1271 | 0.2500 | 0.0982 | 0.0128 | 0.0702 | 0.1058 | 0.1939 | 0.0250 | 0.0085 |
| 277 | 0.0273 | 0.0339 |  | 0.1071 |  | 0.2368 |  | 0.0204 | 0.0125 | 0.0424 |
| 279 | 0.0182 |  | 0.0109 |  | 0.0128 | 0.0614 |  |  | 0.0375 | 0.0678 |
| 281 | 0.0091 |  |  |  |  | 0.0175 |  |  | 0.0125 | 0.0254 |
| 283 |  |  |  |  |  | 0.0088 |  |  |  | 0.0169 |
| 287 |  |  |  |  |  |  |  | 0.0102 |  | 0.0169 |
|  |  |  |  |  |  |  |  |  |  |  |
| *RPGSE34* | TN | WV | NY | NH | ME | QC | NB | NS | NL | MB |
| 255 |  |  |  | 0.0167 |  |  |  |  | 0.0088 |  |
| 257 |  |  |  |  |  |  | 0.0089 |  | 0.0175 |  |
| 259 | 0.0273 |  |  |  |  | 0.0088 | 0.0179 |  | 0.0175 | 0.0172 |
| 261 | 0.0091 |  | 0.0098 |  | 0.0283 | 0.0175 | 0.0089 | 0.0179 | 0.0351 | 0.0172 |
| 263 | 0.0182 | 0.0083 |  |  |  | 0.0263 | 0.0179 |  | 0.0175 | 0.0172 |
| 265 | 0.1000 |  | 0.0392 | 0.0250 | 0.0283 |  | 0.0268 | 0.0179 | 0.0877 | 0.0690 |
| 267 | 0.1364 | 0.0083 | 0.0784 |  | 0.2547 | 0.0351 | 0.1429 | 0.1607 | 0.0526 | 0.0776 |
| 269 | 0.0909 | 0.2167 | 0.0490 | 0.2000 | 0.1226 | 0.1930 | 0.2500 | 0.0625 | 0.0175 | 0.0259 |
| 271 | 0.0455 | 0.0917 |  | 0.0167 | 0.0094 | 0.0877 | 0.0268 | 0.0089 | 0.0439 | 0.0603 |
| 273 | 0.0455 | 0.0250 |  | 0.2333 | 0.0094 | 0.0789 |  | 0.0357 | 0.0877 | 0.0517 |
| 275* | 0.1818 | 0.1000 | 0.0980 | 0.0667 | 0.0755 |  | 0.0268 | 0.0089 | 0.0526 | 0.0948 |
| 277 | 0.2273 | 0.0833 | 0.4706 | 0.2333 | 0.3019 | 0.1053 | 0.2232 | 0.4375 | 0.1579 | 0.0862 |
| 279 | 0.0909 | 0.2500 | 0.2059 | 0.1833 | 0.0943 | 0.1754 | 0.0536 | 0.1250 | 0.1140 | 0.1207 |
| 281 | 0.0273 | 0.1083 | 0.0098 | 0.0167 | 0.0472 | 0.1667 | 0.1339 | 0.0714 | 0.1053 | 0.1552 |
| 283 |  | 0.0500 | 0.0196 | 0.0083 | 0.0189 | 0.0702 |  | 0.0089 | 0.0526 | 0.0862 |
| 285 |  | 0.0333 |  |  |  | 0.0088 | 0.0357 |  | 0.0614 | 0.0603 |
| 287 |  |  |  |  |  | 0.0088 | 0.0179 | 0.0089 | 0.0175 | 0.0086 |
| 289 |  |  |  |  |  | 0.0175 |  |  | 0.0175 | 0.0259 |
| 291 |  |  |  |  |  |  | 0.0089 |  | 0.0351 | 0.0086 |
| 293 |  |  | 0.0196 |  | 0.0094 |  |  | 0.0357 |  | 0.0086 |
| 295R |  | 0.0083 |  |  |  |  |  |  |  |  |
| 297B |  |  |  |  |  |  |  |  |  | 0.0086 |
| 299R |  | 0.0167 |  |  |  |  |  |  |  |  |
|  |  |  |  |  |  |  |  |  |  |  |
| *RPGSE35* | TN | WV | NY | NH | ME | QC | NB | NS | NL | MB |
| 151R |  |  |  |  |  |  | 0.0106 |  |  |  |
| 157R* | 0.0091 |  |  |  |  |  |  |  |  |  |
| 163R |  |  |  |  |  |  |  | 0.0096 |  |  |
| 165R |  |  |  | 0.0167 |  |  |  |  |  |  |
| 167 |  | 0.0106 |  |  |  |  |  |  |  | 0.0593 |
| 169 | 0.0091 |  | 0.0244 | 0.2417 | 0.3558 |  | 0.0638 | 0.1731 | 0.0865 | 0.4576 |
| 171 |  |  | 0.0244 | 0.0667 | 0.3365 | 0.2034 | 0.0745 | 0.0385 | 0.0192 | 0.1780 |
| 173 |  |  |  |  | 0.0192 | 0.1525 |  |  | 0.0192 | 0.0593 |
| 175 |  |  |  |  | 0.0865 | 0.0254 |  | 0.0096 | 0.0192 | 0.1102 |
| 177 |  |  |  |  | 0.0769 | 0.0678 | 0.0106 | 0.0096 | 0.0192 | 0.0339 |
| 179R |  | 0.2128 |  |  |  | 0.0424 | 0.0319 |  |  |  |
| 181 | 0.7000 | 0.5213 | 0.9024 | 0.6583 | 0.0096 |  | 0.7872 | 0.5865 | 0.7500 |  |
| 183 | 0.0091 | 0.0426 | 0.0122 |  | 0.0096 | 0.4831 |  |  |  | 0.0254 |
| 185R* | 0.1273 | 0.1170 | 0.0122 | 0.0167 | 0.0096 | 0.0169 | 0.0106 | 0.0192 |  |  |
| 187R | 0.0091 | 0.0319 |  |  |  |  |  |  |  |  |
| 189 | 0.0091 | 0.0213 |  |  | 0.0577 |  |  |  |  | 0.0339 |
| 191 |  |  | 0.0122 |  | 0.0288 | 0.0085 |  | 0.0192 |  | 0.0424 |
| 193R |  |  |  |  | 0.0096 |  |  |  |  |  |
| 197R* | 0.0455 | 0.0106 |  |  |  |  |  |  |  |  |
| 199* | 0.0455 | 0.0319 |  |  |  |  |  | 0.0288 | 0.0865 |  |
| 201R | 0.0182 |  |  |  |  |  | 0.0106 | 0.1058 |  |  |
| 205R* | 0.0182 |  |  |  |  |  |  |  |  |  |
| 207R |  |  | 0.0122 |  |  |  |  |  |  |  |
|  |  |  |  |  |  |  |  |  |  |  |
| *RPMSA13* | TN | WV | NY | NH | ME | QC | NB | NS | NL | MB |
| 171R |  | 0.0259 |  |  |  | 0.0083 |  |  |  |  |
| 173R |  |  |  |  |  | 0.0250 |  |  |  |  |
| 175R |  |  |  | 0.0083 |  |  |  |  |  |  |
| 177R |  | 0.0862 |  | 0.0083 |  |  |  |  |  |  |
| 179R |  | 0.0172 |  |  | 0.0179 | 0.0083 | 0.0083 |  |  |  |
| 181R |  | 0.0345 | 0.0106 | 0.0917 |  | 0.0083 | 0.0167 |  |  |  |
| 183 | 0.0982 | 0.1379 | 0.0319 | 0.1000 | 0.1071 | 0.1000 | 0.0333 | 0.0254 |  | 0.1321 |
| 185 |  |  | 0.2766 |  | 0.0625 |  | 0.1500 | 0.1186 | 0.0192 | 0.0849 |
| 187 |  |  |  |  |  | 0.0083 | 0.0167 |  | 0.0288 |  |
| 189 | 0.0268 | 0.0086 |  |  |  | 0.0083 | 0.0417 | 0.0424 | 0.0481 | 0.0377 |
| 191 | 0.2857 | 0.2672 | 0.0106 | 0.3500 | 0.0179 | 0.1333 | 0.0167 |  | 0.0385 | 0.0094 |
| 193 | 0.1161 | 0.0862 | 0.2872 | 0.0667 | 0.3482 | 0.2583 | 0.4500 | 0.4068 | 0.1538 | 0.3962 |
| 195 | 0.1161 | 0.0690 | 0.2766 |  | 0.1607 | 0.0917 | 0.0667 | 0.1017 | 0.0769 | 0.0566 |
| 197* | 0.1071 | 0.0690 | 0.0426 | 0.0167 | 0.0268 | 0.0500 | 0.0167 | 0.0593 | 0.0481 | 0.0189 |
| 199 | 0.0536 | 0.0345 | 0.0213 | 0.0583 | 0.0268 | 0.1083 | 0.0750 | 0.0254 | 0.0385 | 0.0377 |
| 201 | 0.0625 | 0.0776 | 0.0213 | 0.0750 | 0.0804 | 0.0500 | 0.0417 | 0.0508 | 0.0385 | 0.0660 |
| 203 | 0.0268 | 0.0086 |  | 0.1667 | 0.0179 | 0.0250 | 0.0167 | 0.0254 | 0.1058 | 0.0377 |
| 205 |  | 0.0172 |  |  | 0.0893 | 0.0417 |  | 0.0424 | 0.1154 | 0.0660 |
| 207 | 0.0625 | 0.0603 |  |  | 0.0268 | 0.0500 | 0.0083 | 0.0254 | 0.0769 | 0.0283 |
| 209 |  |  | 0.0213 | 0.0333 | 0.0179 | 0.0167 | 0.0250 | 0.0763 | 0.1250 | 0.0094 |
| 211 | 0.0089 |  |  | 0.0250 |  | 0.0083 | 0.0083 |  | 0.0577 |  |
| 213B |  |  |  |  |  |  |  |  | 0.0288 |  |
| 215 |  |  |  |  |  |  | 0.0083 |  |  | 0.0189 |
| 235R* | 0.0089 |  |  |  |  |  |  |  |  |  |
| 295R* | 0.0268 |  |  |  |  |  |  |  |  |  |
|  |  |  |  |  |  |  |  |  |  |  |
|  |  |  |  |  |  | nuclear |  |  |  |  |
|  |  |  |  |  |  |  |  |  |  |  |
| Total number of alleles: | | |  |  |  | 139 |  |  |  |  |
| Number of red spruce specific alleles (R): | | | | |  | 36 |  |  |  |  |
| Number of alleles specific for allopatric red spruce: | | | | | | 13 |  |  |  |  |
| Number of alleles specific for sympatric red spruce: | | | | | | 9 |  |  |  |  |
| Number of black spruce specific alleles (B): | | | | |  | 10 |  |  |  |  |
| * - alleles with frequencies showing significant (*p*<0.05) correlation with latitude | | | | | | | | | |  |

**Table S2.** Hardy-Weinberg equilibrium tests.

| Locus |  | TN | WV | NY | NH | ME | QC | NB | NS | NL | MB |
| --- | --- | --- | --- | --- | --- | --- | --- | --- | --- | --- | --- |
| *RPGSE03* | H*_O_* | - | - | 0.1373 | 0.1207 | 0.0400 | 0.0345 | 0.0909 | 0.2105 | 0.4364 | 0.5439 |
|  | H*_E_* | - | - | 0.1508 | 0.2026 | 0.1501 | 0.5193 | 0.3350 | 0.2998 | 0.5910 | 0.5824 |
|  | *p* | - | - | 0.2460 | **0.0153** | **0.0001** | **0.0000** | **0.0000** | **0.0362** | 0.0231 | 0.0788 |
| *RPGSE04* | H*_O_* | 0.8070 | 0.7931 | 0.7619 | 0.8333 | 0.5667 | 0.7167 | 0.6780 | 0.6441 | 0.6207 | 0.6250 |
|  | H*_E_* | 0.7870 | 0.7748 | 0.8256 | 0.8000 | 0.7625 | 0.8499 | 0.7685 | 0.7401 | 0.7106 | 0.6831 |
|  | *p* | 0.7158 | 0.9979 | 0.1915 | 0.0969 | **0.0128** | **0.0039** | **0.0005** | 0.1482 | 0.0256 | 0.2199 |
| *RPGSE05* | H*_O_* | 0.0167 | 0.0167 | 0.2281 | 0.0333 | 0.0500 | 0.0667 | 0.0500 | 0.0678 | 0.0526 | 0.0509 |
|  | H*_E_* | 0.0167 | 0.0167 | 0.4150 | 0.0958 | 0.1679 | 0.2595 | 0.1410 | 0.1299 | 0.1812 | 0.1872 |
|  | *p* | 1.0000 | 1.0000 | **0.0012** | **0.0033** | **0.0001** | **0.0000** | **0.0003** | **0.0027** | **0.0000** | **0.0000** |
| *RPGSE08* | H*_O_* | - | - | 0.0833 | - | 0.0000 | - | - | - | - | - |
|  | H*_E_* | - | - | 0.1108 | - | 0.0650 | - | - | - | - | - |
|  | *p* | - | - | 0.1686 | - | **0.0003** | - | - | - | - | - |
| *RPGSE10* | H*_O_* | 0.5179 | 0.3667 | 0.2093 | 0.5424 | 0.4386 | 0.4746 | 0.3276 | 0.3704 | 0.5088 | 0.5490 |
|  | H*_E_* | 0.5222 | 0.5677 | 0.5551 | 0.5718 | 0.6100 | 0.6894 | 0.6543 | 0.6694 | 0.7303 | 0.6632 |
|  | *p* | **0.0059** | **0.0029** | **0.0000** | **0.0076** | **0.0068** | **0.0007** | **0.0000** | **0.0001** | **0.0000** | 0.1305 |
| *RPGSE29* | H*_O_* | 0.6727 | 0.5593 | 0.5652 | 0.5000 | 0.4872 | 0.6842 | 0.3846 | 0.7143 | 0.4000 | 0.5932 |
|  | H*_E_* | 0.8279 | 0.8647 | 0.7664 | 0.8374 | 0.8868 | 0.8343 | 0.7689 | 0.8300 | 0.8345 | 0.8469 |
|  | *p* | **0.0002** | **0.0000** | **0.0248** | **0.0000** | **0.0000** | **0.0305** | **0.0000** | 0.1243 | **0.0000** | **0.0000** |
| *RPGSE34* | H*_O_* | 0.8546 | 0.7833 | 0.5294 | 0.6333 | 0.6415 | 0.6316 | 0.7321 | 0.6607 | 0.8246 | 0.9138 |
|  | H*_E_* | 0.8721 | 0.8559 | 0.7226 | 0.8184 | 0.8176 | 0.8792 | 0.8494 | 0.7614 | 0.9253 | 0.9222 |
|  | *p* | 0.1516 | 0.1992 | **0.0002** | **0.0013** | **0.0019** | **0.0000** | **0.0224** | 0.0714 | 0.2628 | 0.6767 |
| *RPGSE35* | H*_O_* | 0.4364 | 0.3617 | 0.1220 | 0.2667 | 0.5769 | 0.3729 | 0.3404 | 0.5577 | 0.2115 | 0.4068 |
|  | H*_E_* | 0.4931 | 0.6719 | 0.1861 | 0.5074 | 0.7491 | 0.7006 | 0.3731 | 0.6174 | 0.4251 | 0.7413 |
|  | *p* | 0.4982 | **0.0000** | **0.0037** | **0.0000** | **0.0128** | **0.0000** | 0.1855 | 0.0905 | **0.0000** | **0.0000** |
| *RPMSA13* | H*_O_* | 0.6250 | 0.5517 | 0.7447 | 0.6167 | 0.6429 | 0.7333 | 0.6667 | 0.5763 | 0.5962 | 0.6226 |
|  | H*_E_* | 0.8650 | 0.8793 | 0.7683 | 0.8226 | 0.8271 | 0.8815 | 0.7644 | 0.7988 | 0.9188 | 0.8081 |
|  | *p* | **0.0014** | **0.0000** | 0.1225 | **0.0000** | **0.0021** | **0.0128** | **0.0189** | **0.0000** | **0.0000** | **0.0006** |

**Table S3.** Pairwise Nei's (1972) standard pairwise genetic distance among 10 populations.

|  | TN | WV | NY | NH | ME | QC | NB | NS | NL | MB |
| --- | --- | --- | --- | --- | --- | --- | --- | --- | --- | --- |
| TN | 0 |  |  |  |  |  |  |  |  |  |
| WV | 0.0451 | 0 |  |  |  |  |  |  |  |  |
| NY | 0.0993 | 0.1165 | 0 |  |  |  |  |  |  |  |
| NH | 0.0828 | 0.0638 | 0.1109 | 0 |  |  |  |  |  |  |
| ME | 0.1717 | 0.1353 | 0.1842 | 0.1537 | 0 |  |  |  |  |  |
| QC | 0.2127 | 0.1532 | 0.2893 | 0.1773 | 0.1392 | 0 |  |  |  |  |
| NB | 0.1004 | 0.0932 | 0.0663 | 0.0999 | 0.1324 | 0.2123 | 0 |  |  |  |
| NS | 0.0835 | 0.0924 | 0.0736 | 0.0994 | 0.1046 | 0.1829 | 0.0370 | 0 |  |  |
| NL | 0.2766 | 0.3142 | 0.3250 | 0.3008 | 0.4107 | 0.3403 | 0.2462 | 0.2241 | 0 |  |
| MB | 0.4692 | 0.4624 | 0.5852 | 0.4636 | 0.3298 | 0.2970 | 0.4110 | 0.3380 | 0.1592 | 0 |

**Table S4.** Pairwise *F*_ST_ estimates among 10 populations.

|  | TN | WV | NY | NH | ME | QC | NB | NS | NL | MB |
| --- | --- | --- | --- | --- | --- | --- | --- | --- | --- | --- |
| TN | 0 |  |  |  |  |  |  |  |  |  |
| WV | 0.0330 | 0 |  |  |  |  |  |  |  |  |
| NY | 0.0790 | 0.0867 | 0 |  |  |  |  |  |  |  |
| NH | 0.0649 | 0.0455 | 0.0821 | 0 |  |  |  |  |  |  |
| ME | 0.1197 | 0.0900 | 0.1212 | 0.1011 | 0 |  |  |  |  |  |
| QC | 0.1310 | 0.0926 | 0.1579 | 0.1048 | 0.0742 | 0 |  |  |  |  |
| NB | 0.0782 | 0.0678 | 0.0475 | 0.0724 | 0.0872 | 0.1209 | 0 |  |  |  |
| NS | 0.0632 | 0.0647 | 0.0514 | 0.0695 | 0.0659 | 0.1017 | 0.0211 | 0 |  |  |
| NL | 0.1674 | 0.1741 | 0.1814 | 0.1677 | 0.1924 | 0.1502 | 0.1434 | 0.1271 | 0 |  |
| MB | 0.2355 | 0.2223 | 0.2588 | 0.2221 | 0.1615 | 0.1322 | 0.2041 | 0.1720 | 0.0813 | 0 |

**Table S5.** Individual population level DIYABC comparisons of parameter estimates for admixture scenario.

| Populations | N1 | N2 | N3 | t1 | ra | t2 | NA |
| --- | --- | --- | --- | --- | --- | --- | --- |
| WV MB NB | 4180.00 | 6350.00 | 5610.00 | 566.00 | 0.69 | 5470.00 | 2300.00 |
| NY MB NB | 3560.00 | 5700.00 | 6780.00 | 218.00 | 0.76 | 4630.00 | 2590.00 |
| TN MB NB | 4030.00 | 6540.00 | 5570.00 | 1890.00 | 0.69 | 6480.00 | 3430.00 |
| NY NL NB | 4080.00 | 7220.00 | 5210.00 | 264.00 | 0.77 | 4350.00 | 2150.00 |
| WV NL NB | 4440.00 | 6900.00 | 4870.00 | 414.00 | 0.65 | 4250.00 | 1560.00 |
| TN NL NB | 3710.00 | 7740.00 | 5630.00 | 1590.00 | 0.54 | 5680.00 | 1930.00 |

Estimates of posterior distribution of parameters for the admixture scenario among 6 different combinations of red and black spruce populations. N1 effective population size of southern red spruce population, N2 effective population size of black spruce population, N3 effective population size of admixed population. T1 is the number of generations since recontact and introgression. Gene flow rates (ra and 1-ra) between southern red spruce and black spruce populations into the northern red spruce admixed population. T2 divergence data from the common ancestor in number of generations. NA is the estimated effective population size of the ancestral population. Red spruce populations: TN – Tennessee, WV – West Virginia, NH – New Hampshire, NY – New York, ME – Maine, QC – Quebec, NB – New Brunswick, NS – Nova Scotia. Black spruce: NL –Labrador, MB –Manitoba.


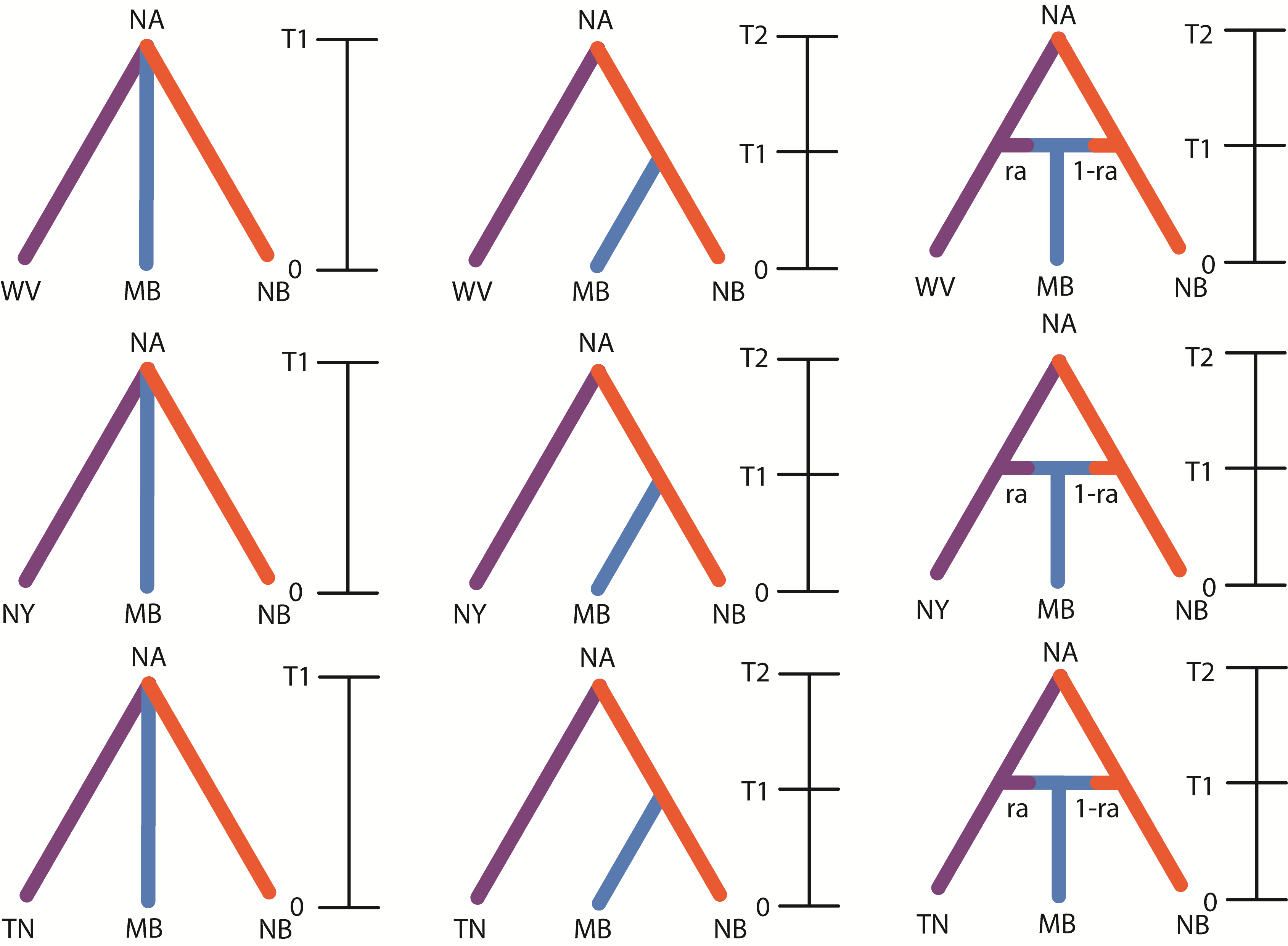

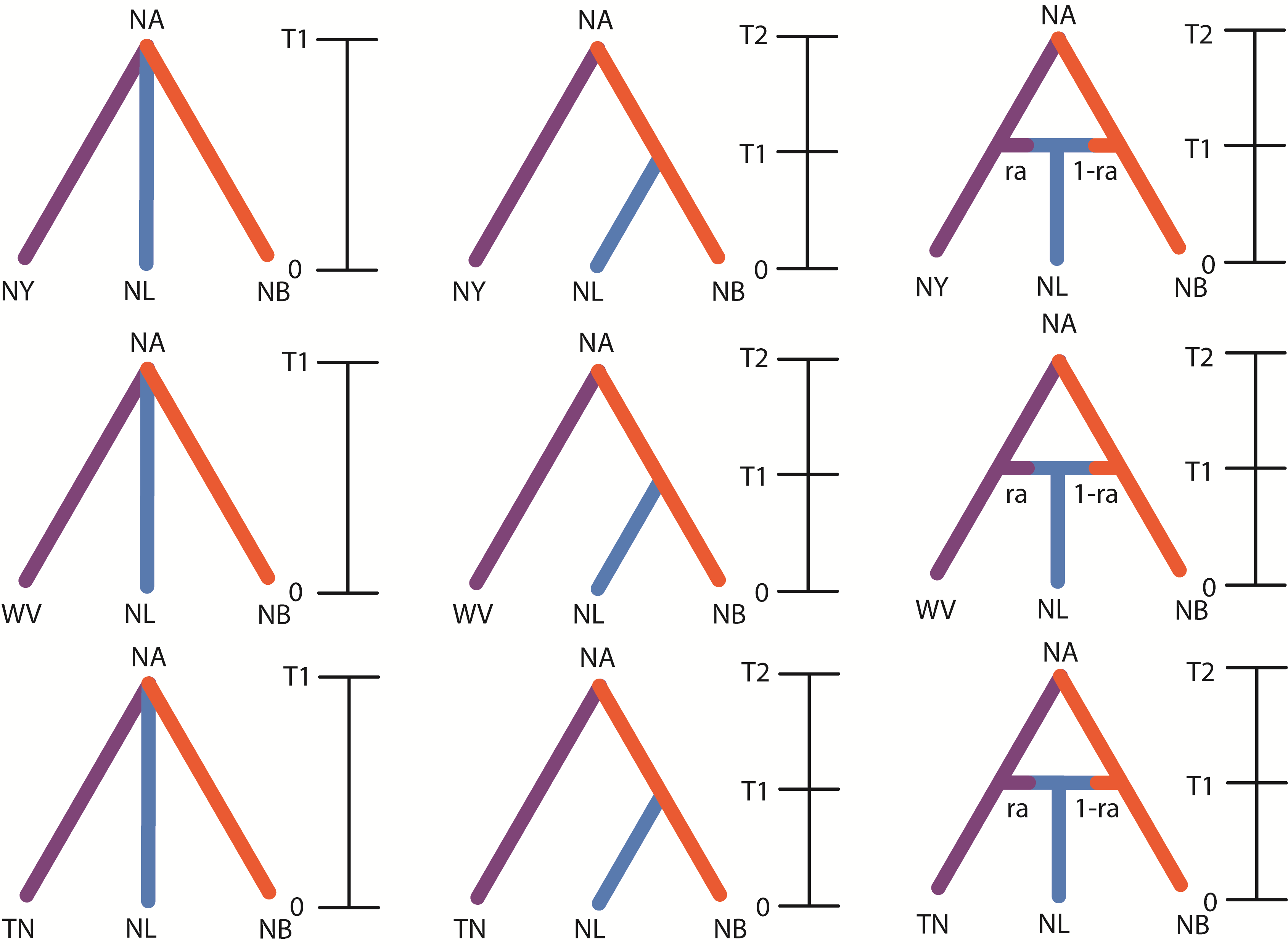


**Fig. S1.** Conceptual models of divergence scenarios among 6 different combinations of red and black spruce populations. T1 is the number of generations since recontact and introgression. Gene flow rates (ra and 1-ra) between southern red spruce and black spruce populations into the northern red spruce admixed population. T2 divergence time from the common ancestor in number of generations. NA is the estimated effective population size of the ancestral population. Red spruce populations: TN – Tennessee, WV – West Virginia, NY – New York, NB – New Brunswick. Black spruce: NL –Labrador, MB –Manitoba. Parameter estimates from the six models are in Table S5.


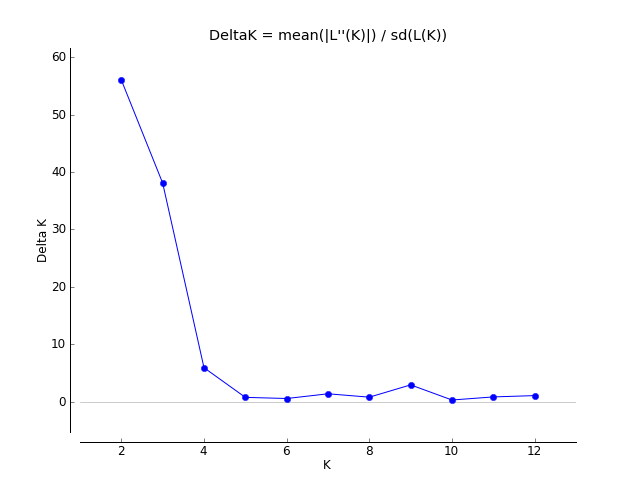


**Fig. S2.** Summary scatterplot of Delta K values for red spruce and black spruce populations testing K= 1 – 14 clusters calculated from the STRUCTURE 2.3.4. (Pritchard et al., 2000) results (**location prior option**) using the Evanno *et al*. method to identify the optimal number of genetic clusters. The Evano method suggested K=2 as the optimal clustering level. At K = 2 the split is between red spruce and black spruce. Two minor peaks were also suggested at at K = 3 and K = 4 indicating genetic substructure within the sampled populations. K = 3 agrees with partitioning of populations between SRS, NRS, and BS while K = 4 represents more complex patterns of isolation, gene flow and introgression in the NRS populations.


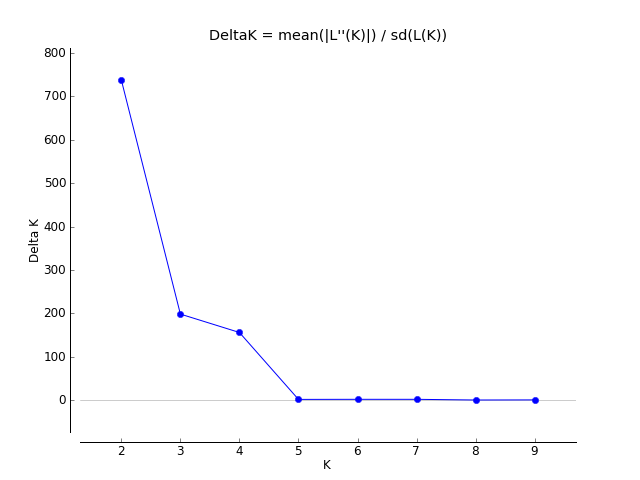


**Fig. S3.** Summary scatterplot of Delta K values for red spruce and black spruce populations testing K= 1 – 14 clusters calculated from the STRUCTURE 2.3.4. (Pritchard et al., 2000) results (**no location prior option**) using the Evanno *et al*. method to identify the optimal number of genetic clusters. The Evano method suggested K=2 as the optimal clustering level. At K = 2 the split is between red spruce and black spruce. Two minor peaks were also suggested at at K = 3 and K = 4 indicating genetic substructure within the sampled populations. K = 3 agrees with partitioning of populations between SRS, NRS, and BS while K = 4 represents more complex patterns of isolation, gene flow and introgression in the NRS populations.


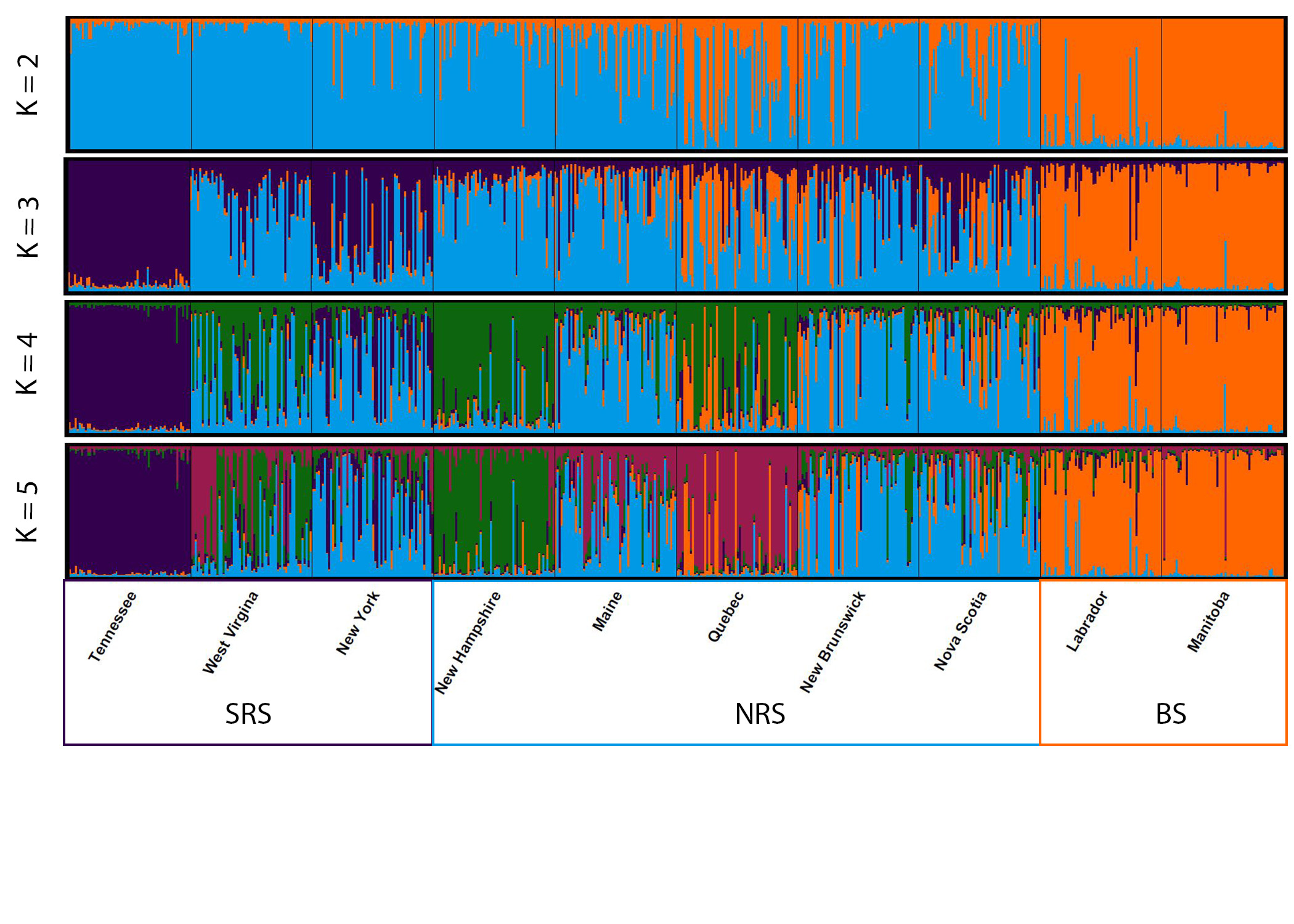


**Fig. S4**. Bayesian clustering results from STRUCTURE 2.3.4. (Pritchard et al., 2000) analysis without location as a prior for K=2 – K=5. The Evanno method (Evanno et al. 2005) suggested K=2 as the optimal clustering level. At K = 2 the split is between red and black spruce. K = 3 through K = 5 represents more complex patterns of isolation, gene flow and introgression in the within and between populations. SRS, Southern allopatric red spruce; NRS, Northern sympatric red spruce; BS, allopatric black spruce.

**Fig. S5**. Unrooted maximum likelihood tree based on nuclear microsatellite markers. Bootstrap support values are given for 1000 replications. Red spruce populations: TN – Tennessee, WV – West Virginia, NH – New Hampshire, NY – New York, ME – Maine, QC – Quebec, NB – New Brunswick, NS – Nova Scotia. Black spruce: NL –Labrador, MB –Manitoba.


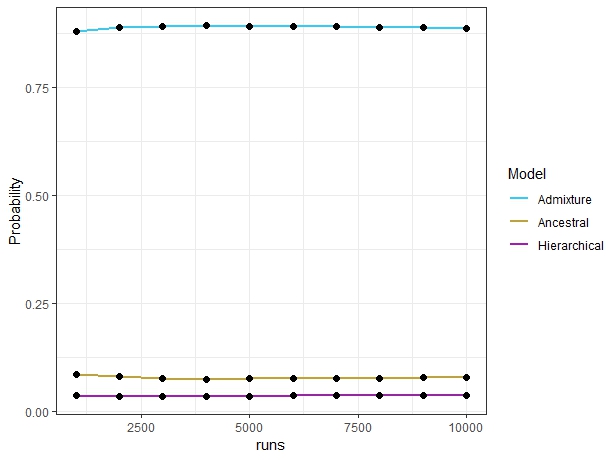


**Fig. S6**. Selection of the most likely scenario was evaluated by comparing the posterior probabilities using logistic regression on 1% of simulated datasets closest to the observed data. The Admixture model was chosen as the most likely scenario based on its higher relative posterior probability compared to the other two models.


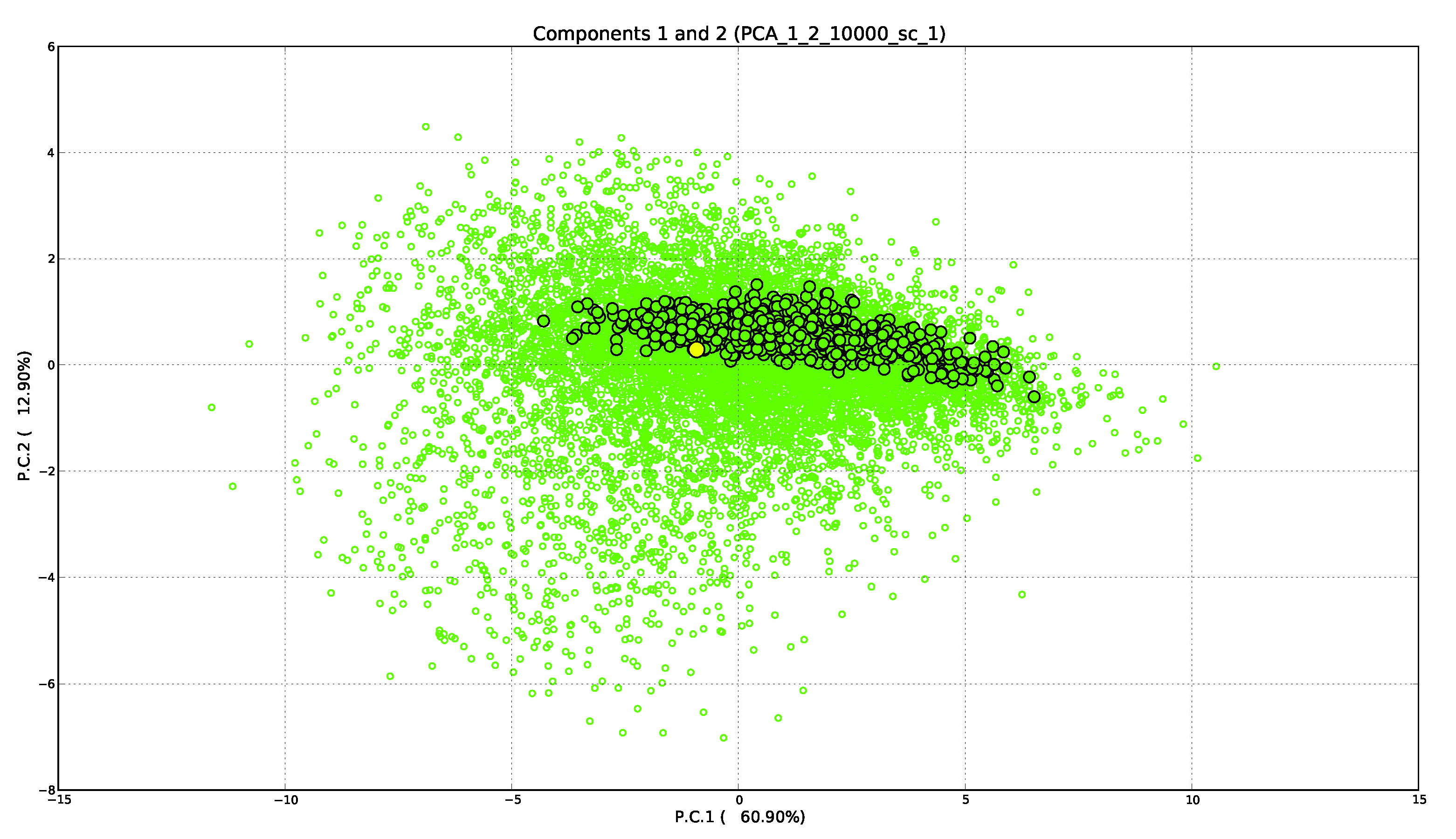


**Fig. S7.** Model checking indicated that the admixture scenario fits well with the data as indicated by the observed data centering (Yellow circle) on the cluster of posterior predictive distribution (medium green circles with black outlines) in the PCA. Small green circles are scenario 1 priors.
